# A new mouse model to study the role of ectopic Nanos3 expression in cancer

**DOI:** 10.1186/s12885-019-5807-x

**Published:** 2019-06-17

**Authors:** Vanessa Andries, Evi De Keuckelaere, Katrien Staes, Tino Hochepied, Joachim Taminau, Kelly Lemeire, Philippe Birembaut, Geert Berx, Frans van Roy

**Affiliations:** 1 VIB-UGent Center for Inflammation Research (IRC), Technologiepark-Zwijnaarde 71, 9052 Ghent, Belgium; 2 Department of Biomedical Molecular Biology, Ghent University, Technologiepark-Zwijnaarde 71, 9052 Ghent, Belgium; 3 Cancer Research Institute Ghent (CRIG), Ghent, Belgium; 4 INSERM UMRS 1250, Department of Biopathology, CHU Maison-Blanche, University Hospital of Reims & University of Reims Champagne-Ardenne, rue Cognacq-Jay 45, 51092 Reims, France

**Keywords:** *NANOS*, Cre-loxP, *ROSA26* locus, Mouse embryogenesis, Lung cancer model

## Abstract

**Background:**

*NANOS3* is a gene conserved throughout evolution. Despite the quite low conservation of Nanos sequences between different organisms and even between Nanos paralogs, their role in germ cell development is remarkably universal. Human Nanos3 expression is normally restricted to the gonads and the brain. However, ectopic activation of this gene has been detected in various human cancers. Until now, Nanos3 and other Nanos proteins have been studied almost exclusively in germ cell development.

**Methods:**

Transgenic mice were generated by targeted insertion of a human Nanos3 cDNA into the *ROSA26* locus. The transgene could be spatiotemporally induced by Cre recombinase activity removing an upstream floxed STOP cassette. A lung tumor model with ectopic Nanos3 expression was based on the lung-specific activation of the reverse tetracycline transactivator gene, in combination with a tetO-CMV promoter controlling Cre expression. When doxycycline was provided to the mice, Cre was activated leading to deletion of *TP53* alleles and activation of both oncogenic KRas^G12D^ and Nanos3. Appropriate controls were foreseen. Tumors and tumor-derived cell cultures were analyzed in various ways.

**Results:**

We describe the successful generation of Nanos3^LSL/−^ and Nanos3^LSL/LSL^ mice in which an exogenous human *NANOS3* gene can be activated in vivo upon Cre expression. These mice, in combination with different conditional and doxycycline-inducible Cre lines, allow the study of the role of ectopic Nanos3 expression in several cancer types. The Nanos3^LSL^ mice were crossed with a non-small cell lung cancer (NSCLC) mouse model based on conditional expression of oncogenic KRas and homozygous loss of p53. This experiment demonstrated that ectopic expression of Nanos3 in the lungs has a significant negative effect on survival. Enhanced bronchiolar dysplasia was observed when Nanos3-expressing NSCLC mice were compared with control NSCLC mice. An allograft experiment, performed with cell cultures derived from primary lung tumors of control and Nanos3-expressing NSCLC mice, revealed lymph node metastasis in mice injected with Nanos3-expressing NSCLC cells.

**Conclusions:**

A new mouse model was generated allowing examination of Nanos3-associated pathways and investigation of the influence of ectopic Nanos3 expression in various cancer types. This model might identify Nanos3 as an interesting target in cancer therapeutics.

**Electronic supplementary material:**

The online version of this article (10.1186/s12885-019-5807-x) contains supplementary material, which is available to authorized users.

## Background

*NANOS3* is one of the three members of the mammalian *Nanos* gene family. The functional role of Nanos proteins has been studied mainly in *Drosophila* and other lower organisms, in which Nanos proteins are essential for anterior-posterior axis polarity, abdomen formation, primordial germ cell migration, germ cell development and survival, and neuronal homeostasis [1–7]. The key role of Nanos proteins in germ cell development has also been confirmed in mammals [8–10]. Both female and male *Nanos3* knockout mice lack germ cells [8]. Nanos3 expression in human embryonic stem cells is similarly essential for maintaining normal germ cell numbers and for the expression of genes required for pluripotency and meiosis [11]. Nanos3 and Nanos proteins in general have been identified principally as post-transcriptional repressors [12, 13]. Nanos proteins exert this activity mainly in concert with their conserved interaction partner, Pumilio [12, 14, 15]. It is this Pumilio association that generally confers mRNA target specificity.

Nanos proteins share a C-terminal zinc-finger domain, which is the only Nanos domain evolutionarily conserved from lower organisms to mammals [16]. The Zf-nanos domain is also the most conserved region between paralogs. Further, vertebrate and some invertebrate Nanos proteins share an additional short N-terminal motif called NOT1 interacting motif (NIM) [12, 16]. The strong conservation of the NIM and Zf-nanos domains among vertebrate Nanos3 proteins points to common interaction partners and functions [12]. The NIM motif is responsible for the interaction of Nanos with the CCR4-NOT deadenylation complex [16–18], while the Zf-nanos domain mediates RNA binding and interaction with the post-transcriptional repressor Pumilio [19, 20]. The mRNA targets of the Nanos/Pumilio complex are mainly recognized by the presence of Nanos response elements (NREs) or Pumilio-binding elements (PBEs), or both, in their 3’UTR sequences [12, 13, 15, 21].

On the other hand, there is emerging evidence for a link between Nanos proteins and tumor progression and cancer [12, 22–27]. Some of these studies revealed ectopic expression of Nanos in tumors [22, 24, 27]. Human Nanos3 was reported to be overexpressed in non-small cell lung cancer (NSCLC), and tumor cells with the highest Nanos3 expression levels were located in cells at the invasion front [27]. In vitro experiments confirmed a role for Nanos3 in the migration and invasion of cultured NSCLC cells [27]. Moreover, the finding that Nanos3 influences epithelial-mesenchymal transition (EMT) by attenuating *CDH1* transcription, leading to decreased E-cadherin expression, and by stimulating vimentin protein expression, point at new mechanisms of EMT regulation [27]. Further, the cBio Cancer Genomics Portal (http://cbioportal.org) indicates that gene amplification is the most common alteration of the *NANOS3* gene in various human cancer types. Despite the putatively important roles of Nanos proteins in malignant cancers, the mechanisms and pathways involved in ectopic Nanos expression are unknown. We present a conditional mouse model that allows ectopic activation of human *NANOS3* in a tissue- and time-specific manner. This model opens interesting avenues to explore a new therapeutic target in cancer. The results of our initial experiments using this mouse model in the context of lung cancer support the idea that *NANOS3* can be considered a tumor promoting gene.

## Methods

### Construction of transgenic Nanos3^LSL^ mice

**Fig. 1:**
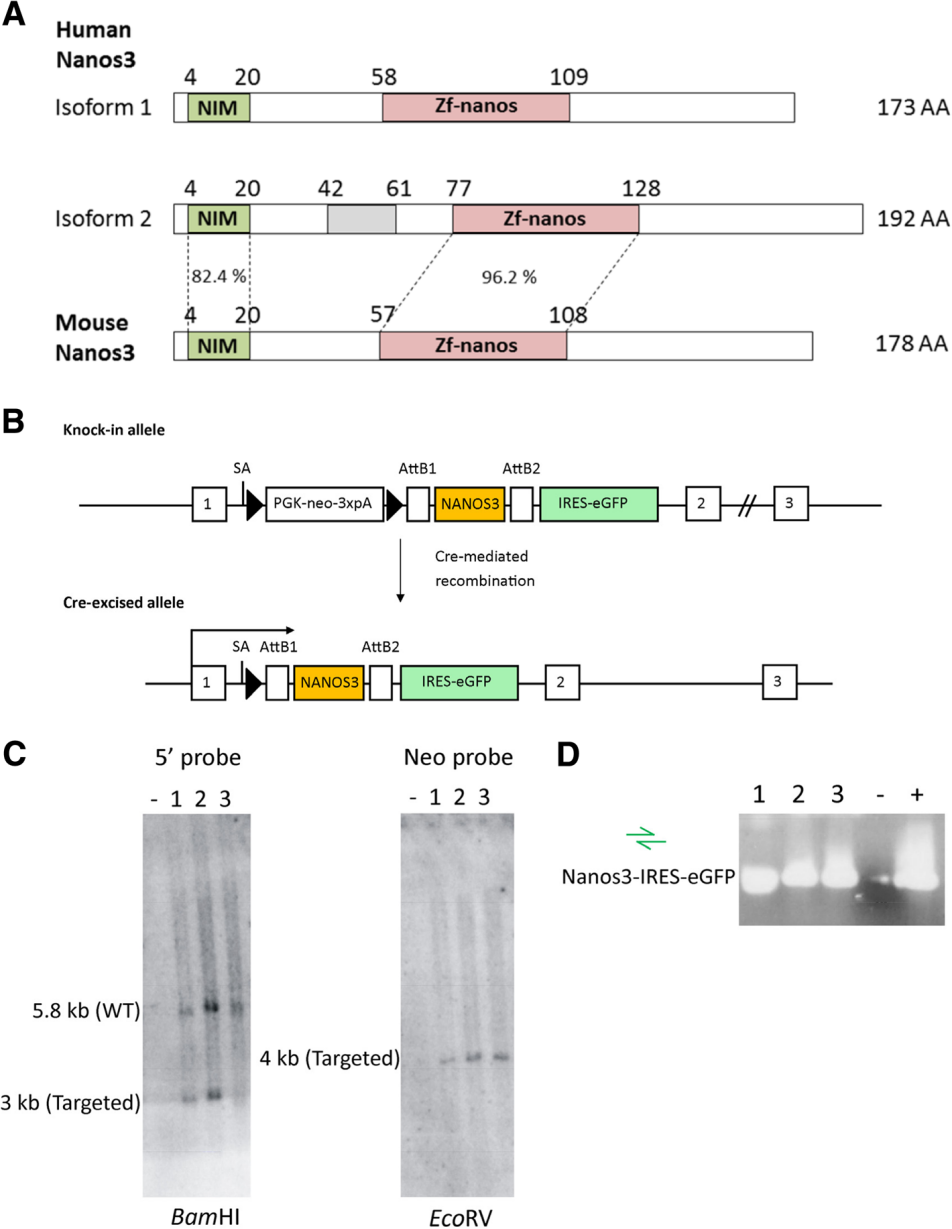
Nanos3 protein domains and sequences and generation of a Nanos3^LSL^ transgene mouse. **a.** Human *NANOS3* is transcribed in two protein-encoding mRNAs. Both translated protein isoforms (173 and 192 amino acids; AA) include the conserved (CCHC)_2_ zinc-finger domain (Zf-nanos) present in all Nanos proteins. In common with all vertebrate and a few invertebrate Nanos proteins, these isoforms have an additional N-terminal NOT1 interacting motif (NIM). The only difference between these two isoforms is an insertion of 19 AA (grey rectangle) corresponding to the retained intron #1 in the correct reading frame (Additional file 1: Figure S1). Such an intron is absent in mouse *Nanos3* transcripts. The percentages represent the sequence identity between the NIM or ZnF motif of human and mouse Nanos3 proteins. **b**. Representation of the knock-in allele found in correctly targeted ES cells. Cre-mediated loxP recombination allows expression of Nanos3 isoform 2 and the IRES-eGFP reporter under control of the *ROSA* promoter. LoxP sites are represented by triangles. SA, splice acceptor. **c**. Southern blot analysis of PCR confirmed targeted ES cells showing an untargeted ES cell line (−) and three correctly targeted ES cell lines (1 to 3). **d**. PCR analysis to confirm the presence of the *NANOS3* and the IRES-eGFP cassette sequences in the three correctly targeted ES cell lines shown in C. A sample without a cDNA template was used as a negative control and a previously tested ES cell line was used as a positive control

Human *NANOS3* cDNA, encoding the longer isoform 2 (Additional file 1: Figure S1), was cloned into a Gateway entry vector (pENTR3C) (Additional file 2: Figure S2). A *ROSA26*-targeting strategy was used as described [28], and detailed in Fig. 1 and Additional file 3: Figure S3. Correctly targeted G4 ES cell clones were identified and validated by PCR and Southern blotting, and used to generate germline-transmitting Nanos3 conditional mice. The primers are listed in Additional file 20: Table S1. ES cells were aggregated with Swiss mouse embryos and transferred into pseudo-pregnant Swiss mice. The resulting Nanos3^LSL/−^ mice were crossed with mice of the C57BL/6 background (Janvier Labs, Saint-Berthevin, France). The Nanos3^LSL/−^ and Nanos3^LSL/LSL^ mice are available to the research community upon request.

### Mouse tumor models

Mice expressing Cre recombinase under control of the rat albumin promoter (Alb-Cre) [29], or the keratin-5 promoter (K5-Cre) [30], were both obtained from the German Research Center for Environmental Health (Neuherberg, Germany). For NSCLC mice on the basis of LSL-KRas^G12D^ and p53^fl/fl^ alleles [31–33], a lung-specific CCSP-rtTA^+/−^;TetO-Cre^+/−^ Tet-on system was used (kindly provided by Dr. A.-K. Perl, Cincinnati Children’s Hospital Medical Center, Ohio, USA), and combined or not with the Nanos3^LSL^ allele. The progenitor p53^fl/fl^ mice were kindly provided by Dr. J. Jonkers (Netherlands Cancer Institute, Amsterdam, The Netherlands). The progenitor LSL-KRAS^G12D^ mice were obtained from the NCI Mouse Repository (Frederick National Laboratory for Cancer Research, Frederick, Maryland, USA). To induce this NSCLC model, the mice were fed normal food pellets supplemented with doxycycline (625 mg/kg, Special Diets Services, Tecnilab-BMI, Someren, The Netherlands) for two weeks starting at the age of two weeks. At appropriate times, tissues were dissected and fixed in 4% paraformaldehyde (PFA) overnight. The lungs were first inflated with 4% PFA and then incubated in 4% PFA for 2 h. For histology, 5-μm paraffin sections were made throughout the entire lung. Genotyping was done by PCR (for primers, see Additional file 20: Table S1) on genomic DNA obtained from tails or ears by standard methods.

All mice were bred and housed at the Vlaams Instituut voor Biotechnologie (VIB, Ghent University) in a specific-pathogen free facility. Mouse experiments were performed in accordance with the Ethics Committee of the Faculty of Science of Ghent University, and were meeting the requirements of Directive 2010/63/EU. All sections of this report adhere to the ARRIVE Guidelines for reporting animal research [34]. A completed ARRIVE guidelines checklist is included as Additional file 21. The Animal Facility Procedures and Licenses of the Inflammation Research Center (Ghent University and VIB, Ghent, Belgium) are overviewed in Additional file 22.

### Immunohistochemistry

Liver, skin and lung sections were deparaffinized, rehydrated and blocked by routine procedures. A citrate buffer was used for antigen retrieval in a 2100 Retriever pressure cooker (PickCell Laboratories, Amsterdam, The Netherlands). Tissues were incubated overnight at 4 °C with antibody against eGFP (Cell Signaling, Danvers, Massachusetts, US; 1:200), Nanos3 (Proteintech, Chicago, USA; 1:200), Vimentin (Gentaur, Kampenhout, Belgium, 1:6000), CC10/CCSP (Millipore, Darmstadt, Germany; 1:4000), SPC (Millipore; 1:4000), E-cadherin (BD Biosciences, New Jersey, US; 1:500), or pan-cytokeratin (Abcam, Cambridge, UK; 1:1800), in PBS containing 5% goat serum and 1% BSA. Slides were incubated with appropriate secondary antibodies (Dako, Glostrup, Denmark) and specific signals were enhanced by use of the ABC-kit (Vector).

### Western blotting

Liver tissues were lysed in Laemmli buffer (50 mM Tris-HCl pH 6.8, 10% glycerol and 2% SDS) supplemented with protease inhibitors (Complete Mini, Roche) for 0.5 h on ice. Lysates were sonicated (Sonics vibra cell™) for 1 min with one-second intervals, after which cell debris was removed by centrifugation. Lung tissues, lung tumor-derived cells and allografts were lysed in a lysis buffer containing 10 mM Tris pH 8.0, 150 mM NaCl and 0.5% NP-40 supplemented with protease inhibitors for 0.5 h at 4 °C. Equal amounts of protein, measured by the DC protein assay kit (Bio-Rad), were separated in a 12% SDS-PAGE gel. Blots were incubated overnight at 4 °C with anti-eGFP antibody (Roche; 1:1000), anti-actin antibody (MP biomedicals; 1:10,000), anti-β-actin antibody (BA3R, ThermoFisher, Massachusetts, USA 1:1000) or G379, a home-made polyclonal Nanos3 antibody raised in rabbits against peptide NH_2_-KKLVRPDKAKTQDTGH-COOH (1:500), or a Nanos3-specific antibody (Proteintech, 1:1000). After incubation with HRP-coupled secondary antibody (Dako), blots were visualized on film (GE Healthcare) using ECL (Thermo Scientific).

### RNA isolation and RT-qPCR

Tissue and cell lysates were homogenized in TRIZOL (Invitrogen) with the Polytron PT 1600E (Kinematica AG) and/or by passing the sample ten times through a 20-gauge needle. The RNeasy Plus Mini kit (Qiagen) was used to isolate total RNA. cDNA was prepared using a SuperScript™ III First-strand Synthesis system (Thermo Fisher). Expression levels of the genes of interest and reference genes were analyzed by real-time quantitative PCR using the SensiFast SYBR No-ROX kit (GC Biotech). Gene expression was normalized to reference genes using qbase+ (Biogazelle) [35]. The primers are listed in Additional file 23: Table S2.

### Measurement of bronchiolar hyperplasia and tumor volumes

H&E sections of the lungs of both control and Nanos3^LSL^ mice of the NSCLC model were scanned with the Axio Scan.Z1 Slide Scanner. To measure the extent of bronchiolar hyperplasia, the surrounding perimeter of bronchioles was manually drawn, and areas were measured using Volocity. The area inside the bronchioles was measured using the magic wand ROI tool of Volocity. The extent of bronchiolar hyperplasia was estimated by subtracting the inside area of the bronchiole from the area of the complete bronchiole. This value was divided by the perimeter of the bronchiole.

Total tumor volumes were estimated using ImageJ 1.51j. A program was written to calculate the tumor percentage making use of a classifier model manually trained on H&E sections of both control and Nanos3-expressing mice, using the Trainable Weka Segmentation plugin in ImageJ.

### Lung tumor-derived cell cultures

Primary lung tumor cell cultures were derived from the lungs of one control (LSL-KRas^G12D^;p53^fl/fl^;CCSP-rtTA^+/−^;TetO-Cre^+/−^) and one Nanos3 (Nanos3^LSL/−^;LSL-KRas^G12D^;p53^fl/fl^;CCSP-rtTA^+/−^;TetO-Cre^+/−^) NSCLC mouse. After dissection, the lungs were incubated in PBS with geneticin (250 μg/ml) for 1 h at room temperature. The lungs were fragmented and dissociated by sterile methods. Dissociation was at 37 °C for 2 to 3 h in DMEM containing 250 μg/ml gentamycin, 0.5% glucose, 0.125 units/ml dispase II, 0.2% collagenase, and 10% FCS. The resulting cell suspension was consecutively run through 70-μm and 40-μm cell strainers, and then centrifuged at 400 x g for 7 min at 4 °C. Cell pellets were suspended in 1 ml of Ammonium-Chloride-Potassium lysing buffer (Lonza) for 5 min. Cells were washed with PBS and seeded in RPMI medium supplemented with 10% FCS and non-essential amino acids.

### Soft agarose assay

Anchorage-independent growth was determined by seeding single-cell suspensions in medium containing 0.35% agarose (Bioline) on top of a 0.76% agarose layer. An additional top-agar layer was added after one week. After 14 days, pictures were taken with a Leica DC300F digital microscope camera using XnView software, and colonies were counted with Volocity. Experiments were performed in duplicate and the mean value was used for further analysis.

### Allograft experiment

Athymic mice (*NMRI*-Foxn1^nu/nu^; Envigo, Horst, The Netherlands) of 5 weeks old were subcutaneously injected with cell cultures derived from the lungs of a control (LSL-KRas^G12D^;p53^fl/fl^;CCSP-rtTA^+/−^;TetO-Cre^+/−^) or a Nanos3 (Nanos3^LSL/−^;LSL-KRas^G12D^;p53^fl/fl^;CCSP-rtTA^+/−^;TetO-Cre^+/−^) NSCLC mouse, using five replicates per cell culture. Per mouse, 2.5 million tumor cells (in 100 μl PBS) were mixed with an equal volume of Matrigel (Corning® Matrigel® Basement Membrane Matrix, VWR) before injection. The length (L), width (W) and height (H) of the tumors were measured twice a week with a caliper till the tumor reached about 1250 mm^3^, or otherwise for a period of maximum 70 days. Tumor volumes were calculated by using the following equation: (π/6) x L x W x H.

### Statistical analysis

The data were analyzed with GraphPad Prism 7. An unpaired Student’s t-test was used to analyze RT-qPCR data. A log-rank (Mantel-Cox) test was performed to analyze the survival curves.

Allograft data were analyzed as repeated measurements using the residual maximum likelihood (REML) approach as implemented in Genstat v18 [36]. Briefly, a linear mixed model with cell cultures, time and cell cultures x time interaction as fixed terms, and subject.time used as residual term, was fitted to the data. Times of measurement were set at equal intervals and an autoregressive correlation structure of either order 1 (AR1) or order 2 (AR2) was selected as best model fit, based on the Akaike Information Coefficient. Significance of the fixed terms and changes in differences across time were assessed by an F-test.

## Results

### Novel mouse model with conditional ectopic expression of human Nanos3

We used an improved transgenesis system, based on cointegration of the transgene of interest with a floxed STOP (LSL) cassette in the Rosa26 locus [28], to efficiently create our transgenic mouse line. A Gateway compatible entry clone containing human *NANOS3* cDNA, encoding the longest Nanos3 isoform 2 (Fig. 1a; Additional file 1: Figure S1 and Additional file 2: Figure S2) but lacking the 3’UTR sequence, was inserted into the previously described pROSA26 destination vector (Additional file 3; Figure S3) [28]. The *NANOS3* cDNA was inserted between a PGK-neo-3xpA (STOP) cassette (in which the neomycin resistance (*neo*^r^) gene is driven by the phosphoglycerine kinase (*PGK*) promoter) and an internal ribosomal entry site (IRES) is placed ahead of an enhanced green fluorescence (eGFP) sequence. This bicistronic vector allows simultaneous expression of the gene of interest and the eGFP reporter protein, but only in cells expressing Cre recombinase. The resultant targeting vector was introduced into F1 hybrid derived (G4) embryonic stem (ES) cells. The primary ES cell colonies were screened by positive selection (neomycin resistance) and negative selection (diphtheria toxin A resistance). Further screening and validation was done by Southern blotting and PCR analyses (Fig. 1c, d). Correctly targeted ES cells were used to generate conditional transgenic Nanos3 mice. In this transgenic line, Cre-dependent expression of *NANOS3* and eGFP is driven by the ROSA26 promoter, which provides a moderate level of expression. We refer to these new transgenic mice as Nanos3^LSL^ mice, namely, Nanos3^LSL/LSL^ (homozygous) and Nanos3^LSL/−^ (heterozygous).

**Fig. 2:**
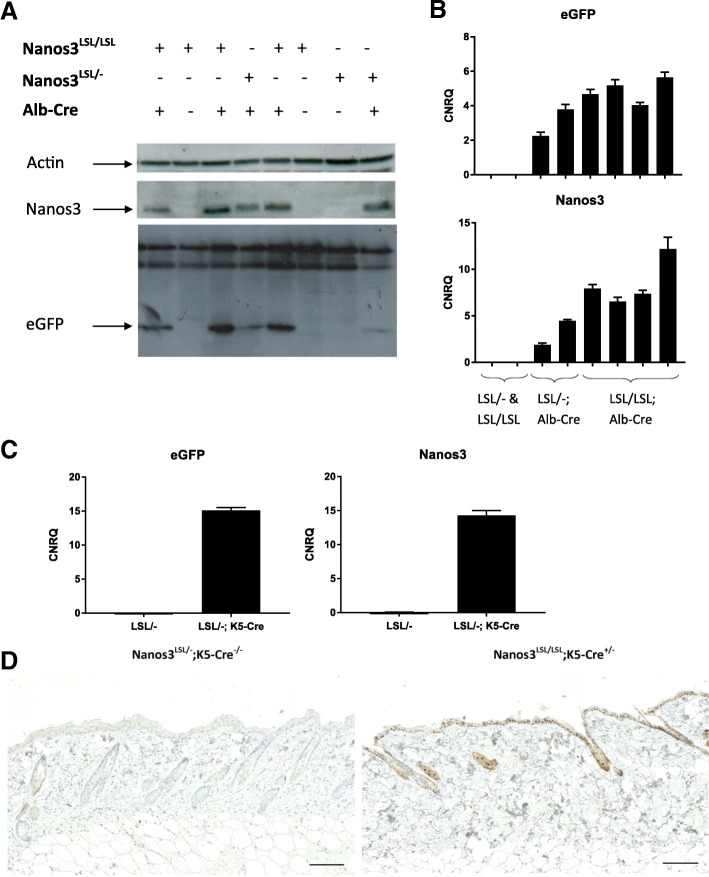
Analysis of the livers of Alb-Cre;Nanos3 mice (**a**, **b**) and skin from K5-Cre;Nanos3 mice (**c**, **d**) with expression of heterozygous or homozygous Nanos3^LSL^ alleles. Western blot (**a**) and RT-qPCR analysis (**b**) of liver lysates from control mice and mice with liver-specific expression of heterozygous or homozygous ectopic Nanos3^LSL^ alleles. **c.** RT-qPCR analysis was done to check for *eGFP* and *NANOS3* RNA expression in RNA lysates of skin from a Nanos3^LSL/−^;K5-Cre^+/−^ mouse and a Nanos3^LSL/−^;K5-Cre^−/−^ mouse. CNRQ, calibrated normalized relative quantity; error bars, SEM; *n* = 3. **b** and **c**. Gene expression was normalized to reference genes (*Tbp* and *Hmbs*) using qbase+ (Biogazelle) [35]. **d.** eGFP expression in skin sections from a Nanos3^LSL/−^;K5-Cre^−/−^ mouse and a Nanos3^LSL/LSL^;K5-Cre^+/−^ mouse was analyzed by immunohistochemical staining. Bars; 100 μm

Depending on the Cre mouse line that is bred with the Nanos3^LSL^ transgenic line, Nanos3 expression is ubiquitous, tissue-specific, or developmental-stage specific. To test the functionality of the expression system, we initially crossed the Nanos3^LSL^ mice with the Sox2-Cre transgenic mouse line [37]. Interestingly, this experiment showed that transgenic expression of Nanos3 in all tissues was embryonically lethal (data not shown). Next, the Nanos3^LSL^ mice were crossed with an albumin-Cre (Alb-Cre) transgenic line [29]. Cre-dependent expression of Nanos3 and eGFP specifically in the liver was confirmed by genotyping, western blotting and RT-qPCR, which proved the reliability of the induction system (Fig. 2a, b). Similarly, we made use of a K5-Cre line [30], to obtain epidermis-specific Cre expression and checked Nanos3 and eGFP expression in the skin by RT-PCR and immunohistochemistry. This confirmed the epidermis-specific expression of Nanos3 in line with the tissue-specific activation of the Cre recombinase (Fig. 2c, d and Additional file 4: Figure S4 for higher magnifications). Both experiments demonstrate the functionality of the conditional Nanos3 expression system, although both the Alb-Cre;Nanos3^LSL^ and the K5-Cre; Nanos3^LSL^ mouse models did not show any evidence of Nanos3-induced pathology.

### Ectopic human Nanos3 expression in a NSCLC model shortens survival

**Fig. 3:**
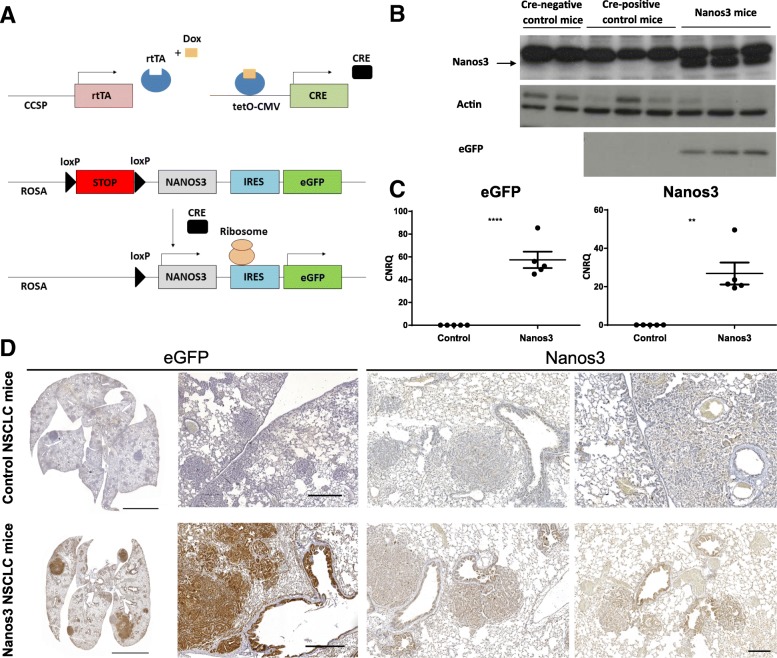
Nanos3 and eGFP expression in the lungs of transgenic mice. **a**. Scheme of inducible Nanos3 expression in the lungs. The club cell secretory protein (CCSP) promoter, active in club cells and type-II alveolar cells, leads to transcription of the reverse tetracycline transactivator gene (rtTA). In the presence of doxycycline (Dox), rtTA binds the tetracycline operator (tetO) in the tetO-CMV promoter, leading to Cre recombinase expression. Cre recombinase mediates recombination between loxP sites and thereby deletes the floxed STOP cassette (LSL) and allows transcription of Nanos3 and enhanced green fluorescent protein (eGFP). IRES: internal ribosomal entry site. To induce transgene expression both control and Nanos3 NSCLC mice were fed doxycycline-containing food and killed about 38 days after Dox induction. **b**. Total lung lysates of two Cre-negative control mice, three Cre-positive control NSCLC mice (LSL-KRas^G12D^;p53^fl/fl^;CCSP-rtTA^+/−^;TetO-Cre^+/−^) and three Nanos3 overexpressing NSCLC mice (Nanos3^LSL/−^;LSL-KRas^G12D^;p53^fl/fl^;CCSP-rtTA^+/−^;TetO-Cre^+/−^) were tested for Nanos3 and the associated eGFP expression by western blotting. Actin was used as a loading control. **c**. RNA prepared from total lung lysates of control and Nanos3-overexpressing mice was used to detect eGFP and Nanos3 transcripts by RT-qPCR. CNRQ, calibrated normalized relative quantity; error bars, SEM; n = 3, **: *P* ≤ 0.01 and ****: *P* ≤ 0.0001. Gene expression was normalized to reference genes (*rpl13A* and *hprt1*) using qbase+ (Biogazelle) [35]. **d**. Lung tumor sections of control NSCLC mice and Nanos3-overexpressing NSCLC mice (Nanos3^LSL/−^;LSL-KRas^G12D^;p53^fl/fl^;CCSP-rtTA^+/−^;TetO-Cre^+/−^) were stained with a GFP- and a Nanos3-specific antibody. Bars: 5 mm for the total lungs, 500 μm for the magnifications of the eGFP staining and 200 μm for the magnifications of the Nanos3 staining

The expression of the *NANOS3* gene in our transgenic mouse model can also be controlled by using the Tet-on system [38]. In that case, expression of the Cre recombinase is controlled by the reverse tetracycline transactivator (rtTA). In the presence of doxycycline (dox), rtTA binds the tetracycline operator (tetO) preceding the Cre recombinase gene, allowing transcription of the latter. Spatiotemporally controlled Cre expression can be obtained by driving rtTA expression by appropriate promoter sequences. We used the Nanos3^LSL^ mice in combination with the established LSL-KRas^G12D^;p53^fl/fl^ lung cancer model [39–41]. In our setup, the rtTA transgene was driven by the rat CCSP promoter (Fig. 3a), which is transcriptionally active in club cells in the bronchioles and in type-II cells in the alveoli. Doxycycline-supplemented food was administered for two weeks, starting at the time of weaning.

Transgenic NSCLC mice in which expression of Nanos3 was induced (Nanos3^LSL/−^;LSL-KRas^G12D^;p53^fl/fl^;CCSP-rtTA^+/−^;TetO-Cre^+/−^) are hereafter referred to as Nanos3 NSCLC mice. Those in which the Nanos3 transgene was not present (LSL-KRas^G12D^;p53^fl/fl^;CCSP-rtTA^+/−^;TetO-Cre^+/−^) are referred to as control NSCLC mice. These Nanos3 NSCLC mice are characterized by ‘ectopic’ expression of Nanos3 in bronchioles and in type-II cells of the alveoli of the lung. In general, ectopic expression involves abnormal gene expression in a cell type, cell tissue type, or developmental stage in which the gene is not usually expressed [42]. In our model, we overexpressed Nanos 3 in the lungs, where it is normally not expressed, to investigate its potential tumor promoting role in lung cancer.

Additionally, in these models, Nanos3-expressing mice express only one exogenous human *NANOS3* allele. Homozygous expression of this transgene was not possible in a straightforward way since the mutant *KRAS* allele was located on chromosome 6 as is the *ROSA* locus where the *NANOS3* transgene was inserted. Lung lysates of Nanos3 and control NSCLC mice were checked for Nanos3 and eGFP expression to assess the functionality of the ROSA26 transgenic expression cassette. As expected, RT-qPCR, western blotting and immunohistochemical analysis confirmed Nanos3 and eGFP expression in the lungs of the Nanos3 NSCLC mice (Figs. 3b-d). A more detailed analysis of eGFP expression, which can be used in our model as a reliable marker for ectopic Nanos3 expression, showed eGFP expression in both adenocarcinoma and bronchiolar neoplasia and confirmed the transcriptional activity of the CCSP promoter in the club cells of the bronchioles and in type-II cells of the alveoli. Interestingly, eGFP expression could be detected in the stromal cells of tumors derived from alveolar tissue, but could not be detected in stromal cells of tumors derived from bronchiolar tissue (Additional file 5: Figure S5).

**Fig. 4:**
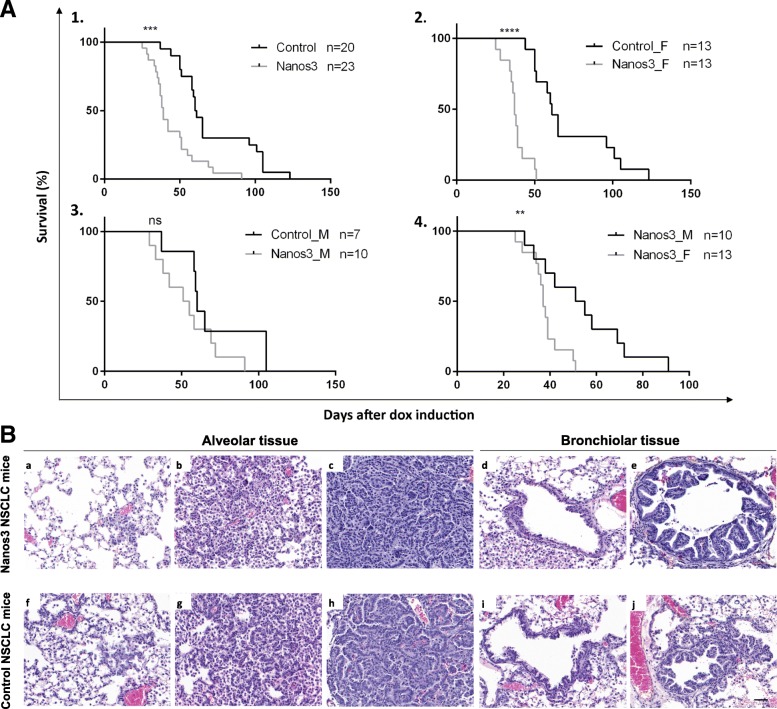
Female mice developing NSCLC die significantly earlier when ectopically expressing Nanos3. **a**. Kaplan-Meier curves for the KRas^G12D^,p53^−/−^ lung cancer model with or without ectopic Nanos3 expression. **A1**. The survival curves of control NSCLC mice compared to Nanos3 NSCLC mice. **A2–4**. Subdivision of the female (_F) and male (_M) control and Nanos3-expressing NSCLC mice. A significant difference was observed in survival between the female control and Nanos3 expressing mice (a2) and between Nanos3-expressing female and male mice (a4). ns: not significant, **: P ≤ 0.01 and ***: *P* ≤ 0.001. **b**. Microscopic images of H&E-stained lung sections from Nanos3-overexpressing (a-e) and control (f-j) NSCLC mice. These sections show alveolar hyperplasia (a and f), atypical adenomatous hyperplasia (b and g) and adenocarcinoma formation (c and h). In the bronchioles, focal hyperplasia (d and i) and papillary hyperplasia (e and j) of bronchiolar epithelial cells is seen. Bar: 50 μm

We found that transgenic control NSCLC mice developed lung tumors resembling those seen in bronchioloalveolar carcinoma (BAC), which in humans was recently named lepidic carcinoma [43]. These tumors remain noninvasive without any stromal reaction. Grelet et al. [27] demonstrated that Nanos3 enhances the invasion rate of cultured NSCLC cells and is involved in EMT regulation. In line with this, we wondered whether Nanos3 overexpression affects tumor progression in vivo. In our NSCLC mouse model, no metastasis was observed regardless of Nanos3 expression. However, mice overexpressing Nanos3 died significantly earlier (Fig. 4a). When comparing male and female control NSCLC mice with, respectively, male and female Nanos3 NSCLC mice, a significant difference was seen only between the female mouse populations. While female control NSCLC mice (*n* = 13) had a median survival of 61 days after dox induction, that of female Nanos3 NSCLC mice (n = 13) was only 37 days after dox induction (*P* < 0.001). On the other hand, male control NSCLC mice (*n* = 7) had a median survival of 60 days after dox induction, whereas male Nanos3 NSCLC mice (*n* = 10) showed a similar median survival of 53 days after dox induction. In both male and female mice, no metastases were observed, and a significant difference between control and Nanos3 NSCLC mice was seen only for female populations. Further experiments were therefore done solely with female mice.

**Fig. 5:**
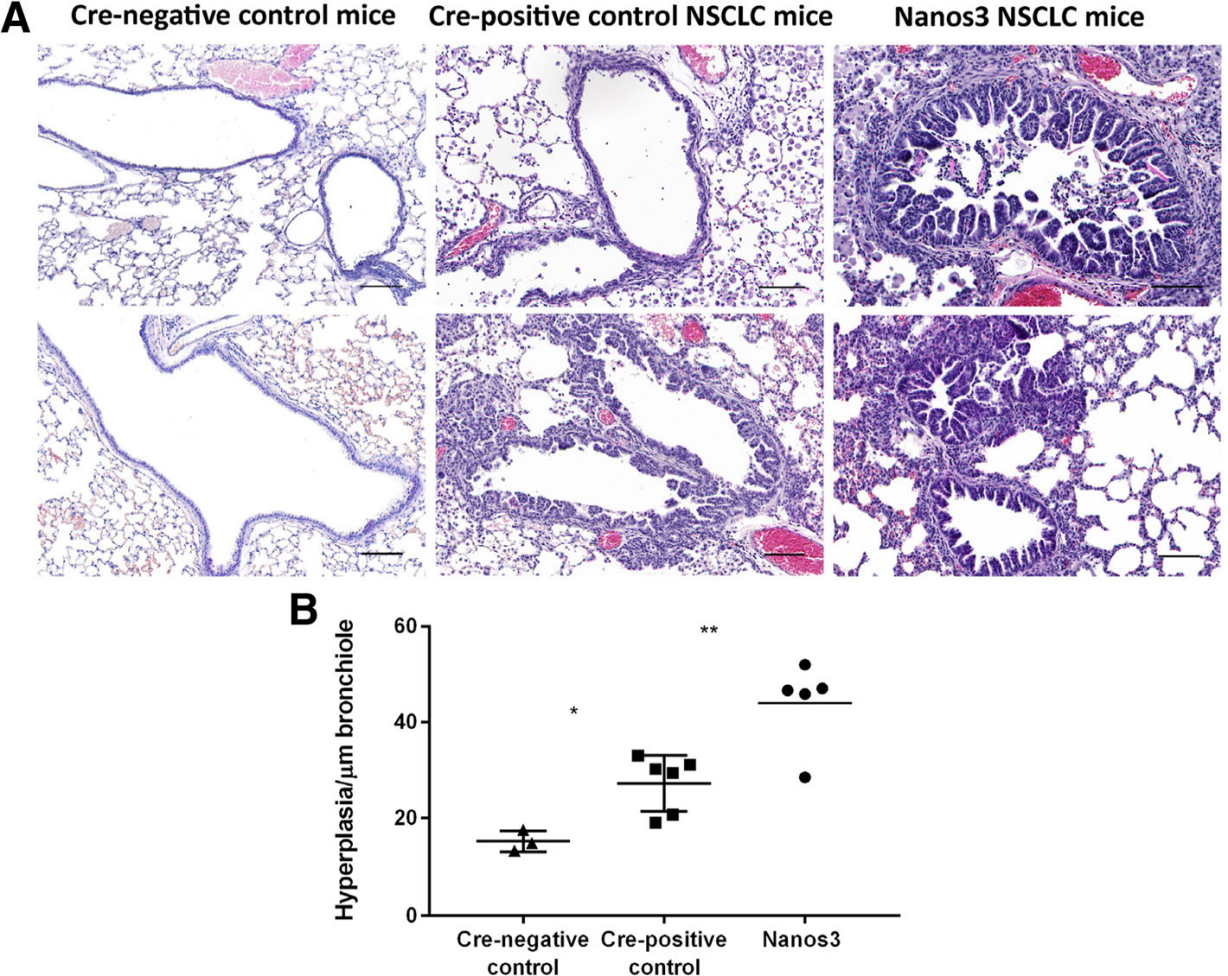
Ectopic Nanos3 expression aggravates bronchiolar hyperplasia in NSCLC mice. **a**. Our mouse model for NSCLC showed bronchiolar hyperplasia compared to Cre-negative control mice. Ectopic Nanos3 overexpression strongly enhanced this bronchiolar phenotype. Representative sections are shown. **b.** Three Cre-negative mice (Nanos3^LSL/−^;LSL-KRas^G12D^;p53^fl/fl^;CCSP-rtTA^+/−^;TetO-Cre^−/−^), five Cre-positive control NSCLC mice (LSL-KRas^G12D^;p53^fl/fl^;CCSP-rtTA^+/−^;TetO-Cre^+/−^) and five Nanos3 NSCLC mice (Nanos3^LSL/−^;LSL-KRas^G12D^;p53^fl/fl^;CCSP-rtTA^+/−^;TetO-Cre^+/−^) were analyzed. The ratio of bronchiolar hyperplasia to the perimeter of the bronchiole was measured in four randomly chosen bronchioles per mouse. The plot shows the average for each mouse. Error bars, SEM; *: *P* ≤ 0.05 and **: *P* ≤ 0.01. Bars: 100 μm

Both control and Nanos3 NSCLC mice were killed about 40 days after dox induction, and their lungs were histologically examined. Different stages of tumor progression were observed (Fig. 4b). Alveolar hyperplasia, premalignant atypical adenomatous hyperplasia (AAH) and adenocarcinoma were observed in the alveolar spaces (Fig. 4b, a-c and f-h; and Additional file 6: Figure S6 for higher magnifications). Focal and papillary hyperplasia were observed in the bronchioles (Fig. 4b, d, e and i, j; and Additional file 7: Figure S7 for higher magnifications). While the lungs of both control and Nanos3 NSCLC mice showed bronchiolar hyperplasia compared to the lungs of tumor-free, Cre-negative control mice, hyperplasia was more prominent in the Nanos3 NSCLC mice (Fig. 5). The lung tumor mass in Nanos3 and control NSCLC mice was measured by image analysis on several H&E slides throughout the entire lungs (see Methods). However, there was no significant difference in tumor mass between the genotypes (Additional file 8: Figure S8).

**Fig. 6:**
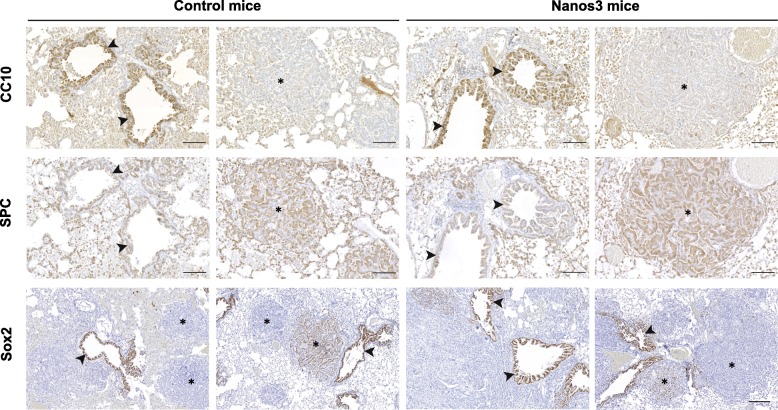
Heterogeneous CC10, SPC and Sox2 staining of bronchioles and adenocarcinomas in NSCLC. Sections of bronchioles (arrowheads) and adenocarcinomas (asterisks) from control and Nanos3 NSCLC mice were stained for CC10, SPC and Sox2. Bars: 100 μm for the CC10 and SPC pictures, and 200 μm for the Sox2 pictures

The bronchioles, including the hyperplastic tissues, stained positive for the Club Cell 10-kDa protein (CC10) and largely negative for Surfactant Protein C (SPC) (Fig. 6; Additional file 9: Figure S9 for CC10 staining, and Additional file 10: Figure S10 for SPC staining). Inversely, the adenocarcinomas were SPC-positive and CC10-negative. The bronchiolar hyperplasias also stained positive for Sox2, an important transcription factor for differentiation of ciliated, club and goblet cells in postnatal bronchioles [44] (Fig. 6). Most of the adenocarcinomas were mainly Sox2-negative but some were completely or partially Sox2-positive (Fig. 6 and Additional file 11: Figure S11). These results indicate that the bronchiolar hyperplastic lesions arose from transformed club cells, while the adenocarcinomas originated mainly from transformed alveolar type-II cells. Several EMT-related immunohistochemical stainings were performed on lung sections of both types of NSCLC mice, with and without Nanos 3 expression. E-cadherin staining showed no evidence of downregulation upon Nanos3 expression in lungs of Nanos3 NSCLC mice (Additional file 12: Figure S12). Moreover, staining for the EMT marker vimentin showed no evidence of upregulation upon Nanos3 expression in this mouse cancer model (Additional file 13: Figure S13). These data indicate that Nanos3 overexpression in this NSCLC model did not influence the expression level of the EMT-related genes, E-cadherin and vimentin.

### The effect of Nanos3 expression on the behavior of cultured lung tumor cells

To further investigate the effect of ectopic Nanos3 expression on tumor progression, cell cultures were derived from primary lung tumors from either a control NSCLC or a Nanos3 NSCLC mouse. These cell cultures will be further referred to as LuTDco and LuTDNa3 cell cultures, respectively.

**Fig. 7:**
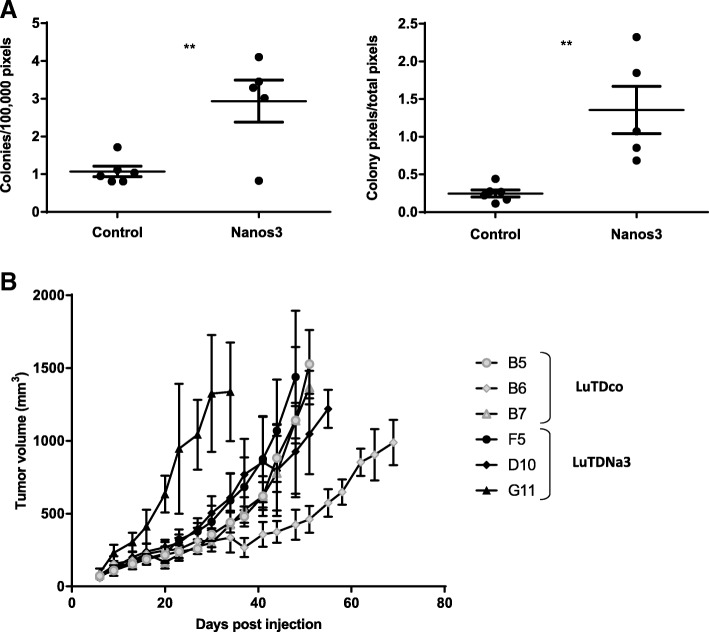
The effects of Nanos3 on the malignant behavior of lung tumor-derived cells. **a.** Soft agar analysis. Cell cultures derived from the lungs of a LSL-KRas^G12D^;p53^fl/fl^;CCSP-rtTA^+/−^;TetO-Cre^+/−^ mouse (control NSCLC) and a Nanos3^LSL/−^;LSL-KRas^G12D^;p53^fl/fl^;CCSP-rtTA^+/−^;TetO-Cre^+/−^ mouse (Nanos3 NSCLC) were grown in a soft agar solution at a density of 10^4^ cells/ml. Parental and N-methyl-N′-nitro-N-nitrosoguanidine (MNNG) transformed HOS (human osteosarcoma) cell cultures were used as a negative and a positive control, respectively. Micrographs were quantified with Volocity. Only colonies bigger than 100 μm^2^ were taken into account. Error bars, SEM; ns: not significant; **: P ≤ 0.01. **b**. Allograft experiment. Five athymic mice were each subcutaneously injected with 2.5 × 10^6^ cells of a culture derived from the lungs of either a control NSCLC or a Nanos3-overexpressing NSCLC mouse. Tumor volumes were measured twice a week. Each graph depicts the average tumor growth curve of mice injected with the same cell culture

Six LuTDco and five LuTDNa3 cell cultures were validated for ectopic Nanos3 and eGFP expression by western blotting and qRT-PCR analysis (Additional file 14: Figure S14). Both experiments showed expression of Nanos3 and eGFP in LuTDNa3 cells, whereas control LuTDco cells did not. A soft agar colony formation assay was then used to measure anchorage-independent growth in vitro. LuTDNa3 cell cultures had a higher anchorage-independent colony formation potential than LuTDco cells (Fig. 7a).

Further, three LuTDco and three LuTDNa3 cell cultures were analyzed in a mouse allograft experiment, using subcutaneous injection into athymic mice (Fig. 7b). In general, ectopic tumors originating from LuTDNa3 and LuTDco cultures grew at similar rates. *NANOS3* and *eGFP* mRNA expression were clearly detected in the ectopic tumors originating from the LuTDNa3 cells but were absent in tumors from the LuTDco cells (Additional file 15: Figure S15).

**Fig. 8:**
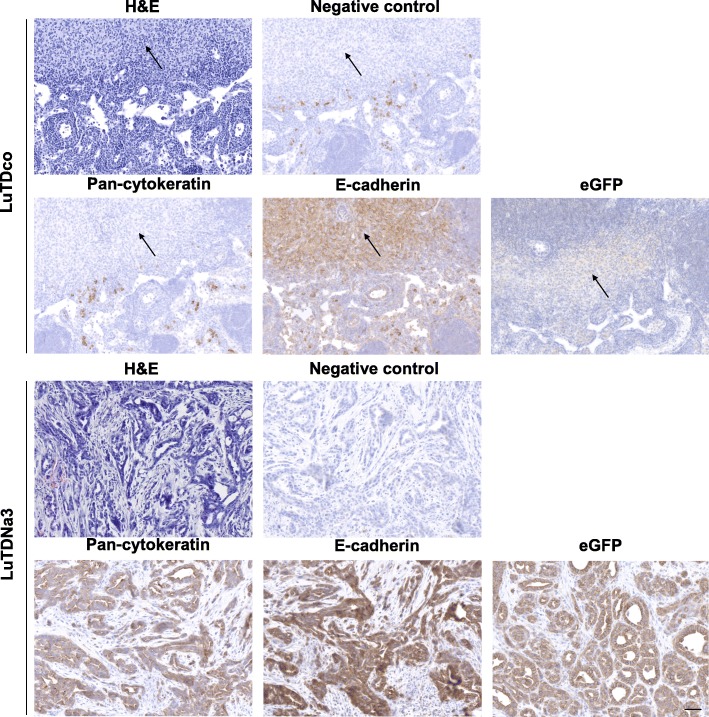
Differentiated metastases in lymph nodes of athymic mice injected with Nanos3-expressing tumor-derived cell cultures. Sections of H&E stained lymph nodes of mice injected with LuTDco or LuTDNa3 cell cultures showed the presence of, respectively, infrequent atypical cells lacking differentiation features (top panels, arrows), and prominent differentiated metastatic lesions (bottom panels). Lymph node sections were stained for E-cadherin and pan-cytokeratin, providing proof for the epithelial origin and the obvious differentiation of the lymph node metastases. Staining of lymph node sections with a GFP-specific antibody confirmed that the nodules in the lymph nodes of the LuTDNa3-injected mice were derived from the transgene-positive primary tumors, as expected. The negative controls represent sections stained with secondary antibodies only. Bars: 50 μm

Small tumor nodules were detected on the lungs of several mice injected subcutaneously with LuTDNa3 or LuTDco cell cultures. However, metastasis formation was not consistently observed in all mice injected with a particular cell culture. Furthermore, no significant difference in lung metastasis was observed in mice subcutaneously injected with either LuTDco or LuTDNa3 cells. In contrast, the axillary lymph nodes of mice subcutaneously injected with the LuTDNa3 cell cultures F5 and D10 were visibly enlarged compared to those from the other mice. For ethical reasons mice injected with LuTDNa3 G11 were sacrificed two to three weeks earlier than those injected with the other LuTDNa3 cell cultures, which might explain why their lymph nodes were not swollen. Histological analysis showed that the axillary lymph nodes of the LuTDNa3-injected mice (F5 and D10), but not those of LuTDco-injected mice, harbored genuine metastases (Fig. 8).

A majority of these lymph node metastases in LuTDNa3-injected mice demonstrated clear evidence of epithelial differentiation with typical histology and strong positivity for pan-cytokeratin and E-cadherin (Additional file 16: Figure S16), coinciding with eGFP positivity (Fig. 8; Additional file 17: Figure S17 for higher magnifications). In contrast, the lymph nodes of mice injected with LuTDco cultures looked largely normal.

To gain more insight into the mechanism why subcutaneous injection of LuTDNa3 cell cultures resulted in lymph node metastasis, we assessed by qRT-PCR the expression level of several migration and invasion markers in the primary lung tumor-derived cell lines LuTDco and LuTDNa3. Intriguingly, this experiment showed no significant differences between LuTDco and LuTDNa3 cell lines in the expression levels of several EMT-related genes, namely *Cdh1*, *Vim*, *Cdh2*, *Fn*, *Snai1* and *Zeb1*, at least not at the mRNA level (Additional file 18: Figure S18). This suggests that other Nanos3-induced pathways are involved in the increased lymph node metastasis of LuTDNa3 cells.

On the contrary, we previously reported that Nanos3 overexpression in human NSCLC cell lines Calu-1 and SK-LU-1 enhanced their invasiveness by up-regulating EMT and wondered whether differences in the level of Nanos3 overexpression could explain this discrepancy between human and mouse lung tumor-derived cells. We thus performed Western blot analysis on lysates of two LuTDco (B5, B6) and two LuTDNa3 cell cultures (D10, F5) and compared the expression levels of Nanos3 with those in lysates of Nanos3-overexpressing human Calu-1 and SK-LU-1 cells. This experiment demonstrated higher normalized Nanos3 expression levels in the mouse tumor derived LuTDNa3 cell lines, compared to the Nanos3-overexpressing human cell lines Calu-1 and SK-LU-1 (Additional file 19: Figure S19), and indicates that higher Nanos3 expression levels do not invariably correlate with higher invasion and migration properties.

## Discussion

We generated a conditional hNanos3-expressing mouse model, Nanos3^LSL^. For this transgenesis, we used a cDNA, encoding the longest isoform of human Nanos3 (192 AA). Compared to the shorter isoform and the mouse Nanos3, this isoform has an insert of 19 AA encoded by a retained intron (Fig. 1a; Additional file 1: Figure S1 and Additional file 2: Figure S2). We chose this human cDNA because this transcript has been annotated in the curated human consensus coding sequence set (CCDS) [45], and because of the reported relevance of ectopic Nanos3 expression in human cancers [12, 27]. In the background of several mouse Cre-lines, the Nanos3^LSL^ mice gave reliable transgene expression fully dependent on the specificity of the Cre recombinase activity. As we found that transgenic expression of Nanos3 in all tissues was embryonically lethal, we opted for the Tet-on system [38], in order to spatiotemporally control Cre expression. This approach is also ideal for combining inducible Nanos3 expression with inducible loss of a tumor suppressor gene or activation of an oncogene, or both, and makes it possible to analyze the influence of ectopic Nanos3 expression in a wide range of cancer models. Previous reports had already linked ectopic expression of Nanos1 and Nanos3 to NSCLC [24, 27]. More specifically, the germline gene *Nanos3* is not expressed in healthy adult somatic tissues, like normal lung tissue, with the exception of testis and brain. However, NANOS3 was found to be expressed in the nucleus and/or cytoplasm of human NSCLC tumor cells independently of their histological subtype. Additionally, stronger staining was frequently observed at the invading front of tumor clusters, especially in squamous cell carcinomas [27]. Therefore, in a first attempt to investigate the in vivo role of ectopic Nanos3 expression in tumor progression, we crossed our Nanos3^LSL^ mouse with a NSCLC mouse model based on LSL-KRas^G12D^ and p53^fl/fl^ alleles [39–41]. Several alternative approaches are of course feasible for lung cancer models, including viral transduction of the Cre gene into the respiratory system [46, 47].

The functions of the Nanos/Pumilio complex in germ cell development, including prevention of apoptosis and inhibition of precocious PGC differentiation [3], seem to be highly conserved from flies to mammals, as elegantly demonstrated by the Saga group [9]. The remarkable migratory behavior of PGCs under the influence of Nanos can be considered an example of non-pathological invasive behavior. Grelet et al. [27] linked Nanos3 to EMT in human NSCLC cell cultures in which Nanos3 overexpression stimulates EMT, whereas its silencing induces mesenchymal–epithelial transition (MET). As invasion and metastasis are the major causes of mortality in cancer patients, it is crucial to understand the molecular mechanisms underlying the successive stages of cancer progression in order to improve prevention, diagnosis and treatment of cancer. We found that ectopic Nanos3 expression in the NSCLC mouse model chosen was associated with significantly shorter survival compared to the genetically matched control mice lacking Nanos3 activity.

The reason for this aggravated pathology is unclear because no metastases were observed in either case. However, the lack of metastasis in this tumor model might be explained by the short median observation time that had to be selected for analysis of the transgenic mice. A mouse lung cancer model based on the combination of a mutant *KRas* allele with a mutant *TP53* allele [48] showed a median survival of mice of 317 days, whereas the median survival for female mice in our NSCLC model was only 37 days. Remarkably, a significant difference between the Nanos3 and control NSCLC mice was seen only in female mice. Our NSCLC models are reminiscent of lepidic carcinoma in humans, which is more common in women [49, 50]. However, this does not offer a full explanation for the sex difference we observed, since the survival of male and female control NSCLC mice was similar. It rather seems that female mice are more prone to Nanos3-induced changes. In general, the incidence and mortality of various cancers are associated with sex-specific disparities [51]. This sex difference in cancer incidence is generally attributed to regulation at the genetic/molecular level and to the effect of sex hormones such as oestrogen and androgen. It might be interesting to investigate whether gene expression levels differ between male and female Nanos3 NSCLC mice, but also the expression levels of Nanos3 interaction partners and Nanos3 mRNA targets should be further investigated in male and female Nanos3 transgenic mice. Such thorough analysis might offer an explanation to the intriguing observed gender preference in our mouse tumor lung model. Additionally, hormonal contribution can also offer an explanation for the differential effects seen between Nanos3 NSCLC males and females. A plausible explanation can be that Nanos3 function is suppressed by androgen receptor signalling or that Nanos3 function is enhanced by oestrogen receptor signalling. However, an initial analysis of GEO profiles did not reveal any significant changes in Nanos3 expression upon oestrogen or testosterone signalling. In conclusion, further studies are needed to provide deeper insight into the observed gender differences in our NSCLC model. Interestingly, a similarly unexplained shorter survival has been reported for female mice in a model for metastatic lung adenocarcinomas based on the combination of a mutant *KRas* allele and a mutant *TP53* allele [48]. Hence, other tumor models using the *Nanos3*^*LSL*^ alleles in combination with activated oncogenes and/or inactivated tumor suppressor genes may increase our knowledge of the in vivo roles of Nanos3 in various cancers.

In our Nanos3^LSL^ model, ectopic Nanos3 expression seemed to affect the club cells in particular, as reflected by significantly more hyperplastic bronchioles compared to the lungs of control NSCLC mice. An important element in cancer progression is the cross-talk between the transformed epithelium and stromal cells. Therefore, it is interesting that tumor-associated stromal cells were eGFP-positive, and therefore most likely Nanos3-positive, in the case of tumors derived from alveolar tissue, whereas this could not be detected in tumors derived from bronchiolar tissue (Additional file 5: Figure S5). This observation needs more thorough investigation, and the use of additional Cre mice in combination with the *Nanos3*^*LSL*^ alleles may reveal important effects of ectopic Nanos3 induction in tumors.

The reason why female Nanos3 NSCLC mice died earlier than the control mice could for instance be ascribed to more severe bronchiolar hyperplasia in Nanos3-expressing females, which might be detrimental to lung function and hence lead to early death. A more advanced stage, bronchiolar papilloma, was not observed in any of the mice we examined. Such progression might have been blocked by the high levels of Sox2 expression observed at the bronchioles [52], which we confirmed in both normal and hyperplastic bronchioles. Alveolar adenocarcinoma formation turned out to be similar in both control and Nanos3-expressing NSCLC mice. Although we have demonstrated in our transgenic mouse models that Nanos3 is potentially oncogenic in NSCLC, our in vivo data do not support the previously proposed role of Nanos3 in E-cadherin suppression and EMT induction in lung cancer [27]. Thus, the exact mechanism controlled by Nanos3 in the NSCLC tumors remains elusive.

Tumor-derived cell cultures from a control and a Nanos3-expressing NSCLC mouse demonstrated anchorage-independent growth, but LuTDNa3 cells generated more and bigger colonies. A subcutaneous allograft experiment with three control LuTDco and three LuTDNa3 cell cultures revealed no reproducible differences in ectopic tumor growth or lung metastasis upon expression of Nanos3. However, large lymph node metastases were only seen in mice injected with LuTDNa3 cells. This observation points to a Nanos3 role in promoting lymph node metastasis. Remarkably, these lymph node metastases showed obvious epithelial tumor differentiation as evidenced by histology, pan-cytokeratin and E-cadherin positivity. Expression analysis for EMT-related genes did not show any significant differences between LuTDco and LuTDNa3 cell lines and therefore suggested that other Nanos3-induced pathways are involved in the increased lymph node metastasis of LuTDNa3 cells. Intriguingly, NANOS3 overexpression in human NSCLC cell lines did enhance their invasiveness by upregulating EMT [27]. However, comparison of Nanos3 protein levels in mouse tumor-derived LuTDNA3 cell lines and Nanos3 overexpressing human NSCLC cell lines did not show higher Nanos3 protein levels in the human cell lines, which might explain the difference in Nanos3-induced EMT between mice and men. In general, many differences occur between mice and men, including documented differential effects of the immune system, life span, metabolism and genomic imprinting [53]. More specifically, Nanos3 is an RNA binding protein that recognizes its mRNA targets by the presence of NRE or PBEs in the 3’UTRs of these target genes. So, small sequence differences between the 3’UTRs of human and mouse transcripts can already change Nanos3 binding properties and should be taken into account when further exploring the observed discrepancy between human tumors and mouse tumor models.

## Conclusions

The here described Nanos3^LSL^ mouse model allows spatiotemporally controlled ectopic expression of human Nanos3. The natural testis/brain-specific expression of this interesting protein and the fact that ectopic expression has been reported in various human cancers makes Nanos3 a potential cancer/testis antigen (CTA) candidate. Our mouse model allows further analysis of the influence of ectopic Nanos3 expression in cancer and in normal tissues. This system can also contribute much to the discovery of physiologically relevant Nanos3 molecular interaction partners and Nanos3 mRNA targets. In addition, it allows further investigation of the effect of Nanos3 on germ cell development in mammals and the pathways involved. Since ubiquitous expression of Nanos3 turned out to be embryonically lethal, the correct localization of Nanos3 protein expression seems to be important in the mammalian embryo, as seen in *Drosophila.* By using the Nanos3^LSL^ mouse model, it will be possible to further investigate the in vivo role of ectopic Nanos3 expression in NSCLC and other cancer types.

## Additional files

Additional file 1: **Figure S1.** The coding DNA sequence and the corresponding Nanos3 protein sequence of the human *NANOS3* gene. After splicing, this gene is transcribed into two isoforms. The first intron (first red horizontal arrow) is retained in the transcript encoding isoform 2, and encodes an in-frame peptide. (DOC 1602 kb)

Additional file 2: **Figure S2.** Part of the entry vector sequence containing the AttL sites and the cDNA sequence of the *Nanos3* entry clone used to make the Nanos3 transgenic mice. (DOC 1392 kb)

Additional file 3: **Figure S3.** Generation by homologous recombination into the *ROSA26* locus of a transgenic mouse allowing conditional ectopic expression of a human *NANOS3* allele. The Gateway® Nanos3 entry clone was recombined with the *ROSA26* destination vector (pROSA26-DV1) [28], using LR clonase. The targeting vector was replicated in bacteria, subsequently linearized (*Pvu*I) and electroporated in ES cells, where homologous recombination with the wild type (WT) *ROSA26* locus took place. Correctly targeted ES cells were selected (resistance to geneticin [neomycin-resistant cells] and diphtheria toxin A [DTA]) and screened by PCR and Southern blot analyses. The blue (G1 and G2) and green (Nanos3_F and Nanos3_R) arrows represent the sequencing primers used (Fig. 1d; Additional file 20: Table S1). The black and red rectangles represent the 5′ probe and neo probe, respectively, used for Southern blot analysis (Fig. 1c). The expected band sizes after genomic DNA digestion of the WT or knock-in allele with the corresponding restriction enzymes are indicated by the double-headed arrows. Cre-mediated loxP recombination allows expression of Nanos3 and the IRES-eGFP reporter under control of the *ROSA26* promoter. The resulting mice were genotyped using the primers represented by black (Rosa_F, Rosa_R1 and Rosa_R2) and green arrows (Nanos3_F and Nanos3_R) (Additional file 20: Table S1). LoxP sites are represented by triangles. SA, splice acceptor site. (DOC 374 kb)

Additional file 4: **Figure S4.** Epidermis-specific expression of the *Nanos3* transgene. eGFP expression in skin sections from a Nanos3^LSL/−^;K5-Cre^−/−^ mouse and a Nanos3^LSL/LSL^;K5-Cre^+/−^ mouse was analyzed by immunohistochemical staining. Bottom panels show the same fields as top panels, but with increased magnification. Bars, 100 μm. (PDF 3030 kb)

Additional file 5: **Figure S5.** eGFP expression in lungs of control and Nanos3 NSCLC mice. Sections of adenocarcinomas (top panels) and bronchioles (bottom panels) from control and Nanos3 NSCLC mice were stained for eGFP. Expression is evident in both alveolar and bronchiolar hyperplasia of Nanos3 NSCLC mice. Arrows point at stromal cells of an adenocarcinoma tumor. From left to right, panels correspond to images with increased magnification. Bars, 50 μm. (PDF 7230 kb)

Additional file 6: **Figure S6.** Microscopic images of H&E-stained lung sections from control and Nanos3 NSCLC mice show different stages of tumor progression in the alveolar spaces. Alveolar hyperplasia, premalignant atypical adenomatous hyperplasia and adenocarcinoma were observed in the alveolar spaces of both control and Nanos3 NSCLC mice. Panels correspond to increasing magnification from left to right. Bars, 50 μm. (PDF 8592 kb)

Additional file 7: **Figure S7.** Microscopic images of H&E-stained lung sections from control and Nanos3 NSCLC mice show different stages of tumor progression in the bronchiolar tissue. Focal and papillary hyperplasia were observed in the bronchioles of both control and Nanos3 NSCLC mice. Panels correspond to increasing magnification from left to right. Bars, 50 μm. (PDF 6768 kb)

Additional file 8: **Figure S8.** The tumor percentage of the lungs is comparable in control and Nanos3 NSCLC mice. Five H&E sections throughout the complete lungs were used to measure the tumor mass by scanning followed by appropriate image analysis as detailed in Methods. Quantification was done with ImageJ. Error bars, SEM. (PDF 9 kb)

Additional file 9: **Figure S9.** CC10 expression in adenocarcinomas and bronchioles of control and Nanos3 NSCLC mice. CC10 staining of lung sections of adenocarcinomas (top panels) and bronchioles (bottom panels) from control and Nanos3 NSCLC mice. Panels correspond to increasing magnification from left to right. Bars, 50 μm. (PDF 6460 kb)

Additional file 10: **Figure S10.** SPC expression in adenocarcinomas and bronchioles of control and Nanos3 NSCLC mice. SPC staining of lung sections of adenocarcinomas (top panels) and bronchioles (bottom panels) from control and Nanos3 NSCLC mice. Panels correspond to increasing magnification from left to right. Bars, 50 μm. (PDF 6333 kb)

Additional file 11: **Figure S11.** Sox2 expression in adenocarcinomas and bronchioles of control and Nanos3 NSCLC mice. Sox2 staining of lung sections of adenocarcinomas (top panels) and bronchioles (bottom panels) from control and Nanos3 NSCLC mice. Panels correspond to increasing magnification from left to right. Bars, 50 μm. (PDF 6975 kb)

Additional file 12: **Figure S12.** E-cadherin expression in the bronchioles and adenocarcinomas of NSCLC mice. **A.** E-cadherin staining of lung sections from control (LSL-KRas^G12D^;p53^fl/fl^;CCSP-rtTA^+/−^;TetO-Cre^+/−^) and Nanos3 (Nanos3^LSL/−^;LSL-KRas^G12D^;p53^fl/fl^;CCSP-rtTA^+/−^;TetO-Cre^+/−^) NSCLC mice. Bar, 200 μm. **B.** Both normal and hyperplastic bronchioles stained positive for E-cadherin. Bar, 50 μm. (DOC 12361 kb)

Additional file 13: Figure S13. Vimentin expression in adenocarcinomas and bronchioles of control and Nanos3 NSCLC mice. Vimentin staining of lung sections of adenocarcinomas (top panels) and bronchioles (bottom panels) from control and Nanos3 NSCLC mice showed similar vimentin expression patterns for control and Nanos3 NSCLC mice. Panels correspond to increasing magnification from left to right. Bars, 50 μm. (PDF 6633 kb)

Additional file 14: **Figure S14.** Nanos3 and eGFP expression of primary lung cancer cell cultures. Primary cell cultures derived from the lungs of a control NSCLC mouse (LuTDco) and a Nanos3 overexpressing NSCLC mouse (LuTDNa3) were tested for Nanos3 and eGFP expression by western blotting **(A)** and RT-qPCR **(B)**. Actin was used as a loading control for western blot analysis. CNRQ, calibrated normalized relative quantity, error bars, SEM; *n* = 3. Gene expression was normalized to reference genes (*rpl13A*, *ywhaz* and *sdha*) using qbase+ (Biogazelle) [35]. (DOC 654 kb)

Additional file 15: **Figure S15.** Analysis of eGFP and NANOS3 mRNA and protein expression in ectopic tumors (allografts) from control and Nanos3-expressing lung tumor-derived cell cultures. Ectopic tumors were dissected from athymic mice injected subcutaneously with cultured primary lung cancer cells derived from either a LSL-KRas^G12D^;p53^fl/fl^; CCSP-rtTA^+/−^;TetO-Cre^+/−^ mouse (LuTDco) or a Nanos3^LSL/−^;LSL-KRas^G12D^;p53^fl/fl^;CCSP-rtTA^+/−^;TetO-Cre^+/−^ mouse (LuTDNa3). **A.** RNA lysates were made from part of the ectopic subcutaneous tumors originating from LuTDco or LuTDNa3 cell cultures. Each dot represents an ectopic tumor from an athymic mouse injected with these cell cultures. CNRQ, calibrated normalized relative quantity. Error bars, SEM; ns: not significant, ****: *P* ≤ 0.0001. Gene expression was normalized to reference genes (*rpl13A*, *ywhaz* and *sdha*) using qbase+ (Biogazelle) [35]. **B.** Protein lysates from the allografts from the injected mice (M0 to M4) were checked for Nanos3 and eGFP expression by western blotting. Actin was used as a loading control. (DOC 768 kb)

Additional file 16: **Figure S16.** Epithelial differentiation in lymph node metastasis of mice subcutaneously injected with LuTDNa3 cell cultures. Lymph node sections were stained for E-cadherin (**A**) and pan-cytokeratin (**B**), providing proof for the epithelial origin and the obvious differentiation of the lymph node metastases. Panels correspond to increasing magnification from left to right. Bars, 50 μm. (PDF 6514 kb)

Additional file 17: **Figure S17.** Lymph node metastasis of mice subcutaneously injected with LuTDNa3 cell cultures. **A.** Sections of H&E stained lymph nodes of mice injected with LuTDco or LuTDNa3 cell cultures showed the presence of, respectively, infrequent atypical cells lacking differentiation features (top panels), and prominent differentiated metastatic lesions (bottom panels). **B.** Lymph node sections were stained for eGFP and this confirmed that the metastatic lesions in the lymph nodes of the LuTDNa3-injected mice were derived from the transgene-positive primary tumors, as expected. Panels correspond to increasing magnification from left to right. Bars, 50 μm. (PDF 7298 kb)

Additional file 18: **Figure S18.** EMT is not involved in the increased lymph node metastasis by LuTDNa3 cells. **A.** Expression analyses of EMT-related genes by qRT-PCR of transcripts in primary lung tumor-derived cell lines LuTDco and LuTDNa3. This experiment revealed no significant differences between LuTDco and LuTDNa3 cell lines for the expression levels of the following genes, *Cdh1*, *Vim*, *Cdh2*, *Fn*, *Snai1* and *Zeb1*. **B.** Expression analyses of *eGFP* and *Nanos3* by qRT-PCR in primary lung tumor-derived cell lines LuTDco and LuTDNa3 showed specific eGFP and Nanos3 expression in LuTDNa3 cell lines. CNRQ, calibrated normalized relative quantity, error bars, SEM, ***: *P* ≤ 0.001 and ****: P ≤ 0.0001 Gene expression was normalized to reference genes (*eef1a* and *hmbs*) using qbase+ (Biogazelle) [35]. (PDF 1358 kb)

Additional file 19: **Figure S19.** Comparison of Nanos3 expression levels in lung tumor-derived mouse cell lines and established human lung cancer cell lines. **A.** Protein levels of Nanos3 were detected by western blotting. Top and middle panel represent lower and higher exposure time, respectively, for Nanos3 detection. The Nanos3-specific bands are indicated by the arrows. Actin expression acted as a loading control (bottom panel). **B.** Quantification of Nanos3 levels in the blot of (A), normalized against actin signals. (PDF 1411 kb)

Additional file 20: **Table S1**. A list of the primers used for genotyping. (DOCX 14 kb)

Additional file 21 Completed ARRIVE Guidelines Checklist. (PDF 1093 kb)

Additional file 22: Animal Facility Procedures and Licenses of the Inflammation Research Center, Ghent University and VIB, Ghent, Belgium. (PDF 2414 kb)

Additional file 23: **Table S2**. A list of the RT-qPCR primers used. (DOCX 14 kb)

## Data Availability

All data generated or analysed during this study are included in this published article and its supplementary information files. The Nanos3^LSL/−^ and Nanos3^LSL/LSL^ mice are available from the corresponding author on reasonable request.

## Abbreviations

AAH: Atypical adenomatous hyperplasia
Alb-Cre: albumin Cre
BAC: Bronchioloalveolar carcinoma
CC10: Club cell 10-kDa protein
CCDS: Consensus coding sequence set
CCSP: Club cell secretory protein
CNRQ: Calibrated normalized relative quantity
control NSCLC mice: LSL-KRas^G12D^;p53^fl/fl^;CCSP-rtTA^+/−^;TetO-Cre^+/−^ mice
Cre-negative mice: Nanos3^LSL/−^;LSL-KRas^G12D^;p53^fl/fl^;CCSP-rtTA^+/−^;TetO-Cre^−/−^ mice
CTA: Cancer/testis antigen
dox: Doxycycline
eGFP: enhanced green fluorescence protein
EMT: Epithelial-mesenchymal transition
ES: Embryonic stem
HOS: Human osteosarcoma
IRES: Internal ribosomal entry site
K5-Cre: keratin-5 Cre
LSL: floxed STOP cassette
LuTDco: LSL-KRas^G12D^;p53^fl/fl^;CCSP-rtTA^+/−^;TetO-Cre^+/−^ tumor-derived primary culture
LuTDNa3: Nanos3^LSL/−^;LSL-KRas^G12D^;p53^fl/fl^;CCSP-rtTA^+/−^;TetO-Cre^+/−^ tumor-derived primary culture
MET: mesenchymal–epithelial transition
MNNG: N-methyl-N′-nitro-N-nitrosoguanidine
Nanos3 NSCLC mice: Nanos3^LSL/−^;LSL-KRas^G12D^;p53^fl/fl^;CCSP-rtTA^+/−^;TetO-Cre^+/−^ mice
neo^r^: neomycin resistance
NIM: NOT1 interacting motif
NRE: Nanos response elements
NSCLC: non-small cell lung cancer
PBE: Pumilio-binding element
PFA: Paraformaldehyde
PGK: Phosphoglycerine kinase
REML: Residual maximum likelihood
RT-qPCR: quantitative reverse transcription polymerase chain reaction
rtTA: reverse tetracycline transactivator
SEM: Standard error of mean
SPC: Surfactant Protein C
tetO: tetracycline operator
UTR: Untranslated region
Zf-nanos: (CCHC)_2_ zinc-finger domain of Nanos proteins
