# Supplementary figures and images for: A new mouse model to study the role of ectopic Nanos3 expression in cancer
Source: BMC Cancer. 2019 Jun 17;19:598. doi: 10.1186/s12885-019-5807-x (PMC6580527; doi:10.1186/s12885-019-5807-x)

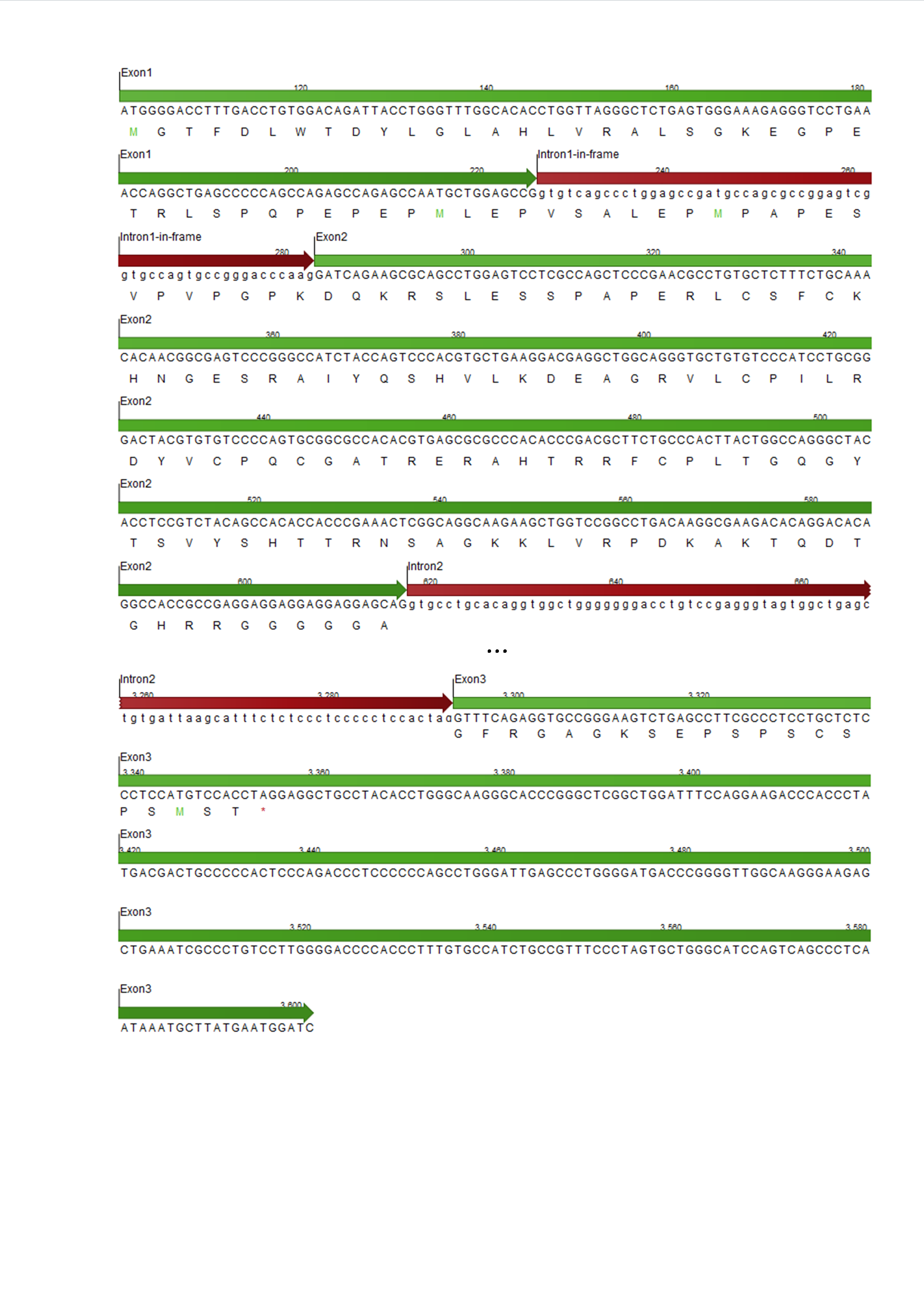


Figure S1 - Andries *et al.*

Supplement: Supplementary file 1 — Figure S1. The coding DNA sequence and the corresponding Nanos3 protein sequence of the human NANOS3 gene. After splicing, this gene is transcribed into two isoforms. The first intron (first red horizontal arrow) is retained in the transcript encoding isoform 2, and encodes an in-frame peptide. (DOC 1602 kb) [file 12885_2019_5807_MOESM1_ESM.doc]

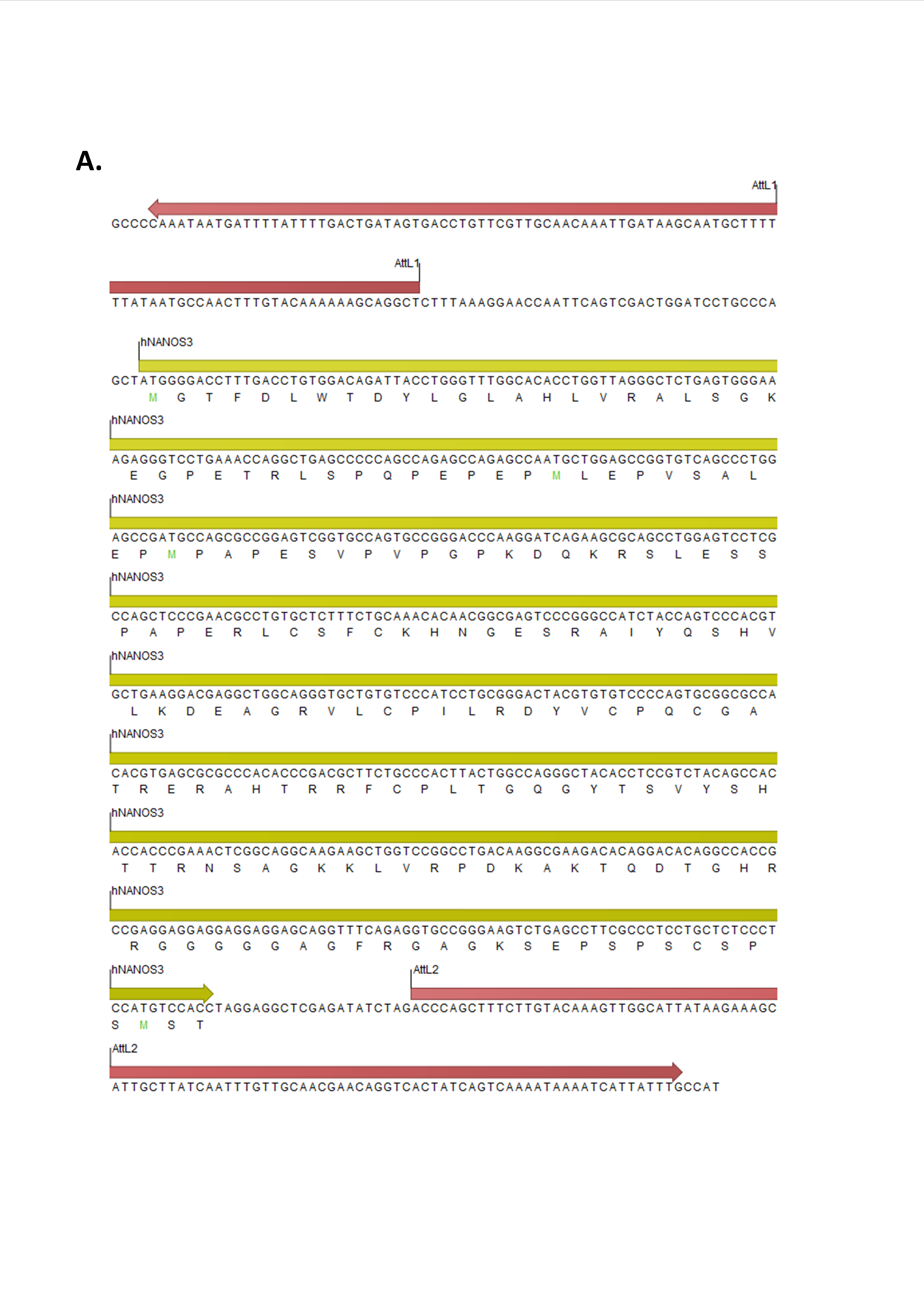


Figure S2 - Andries *et al.*

Supplement: Supplementary file 2 — Figure S2. Part of the entry vector sequence containing the AttL sites and the cDNA sequence of the Nanos3 entry clone used to make the Nanos3 transgenic mice. (DOC 1392 kb) [file 12885_2019_5807_MOESM2_ESM.doc]

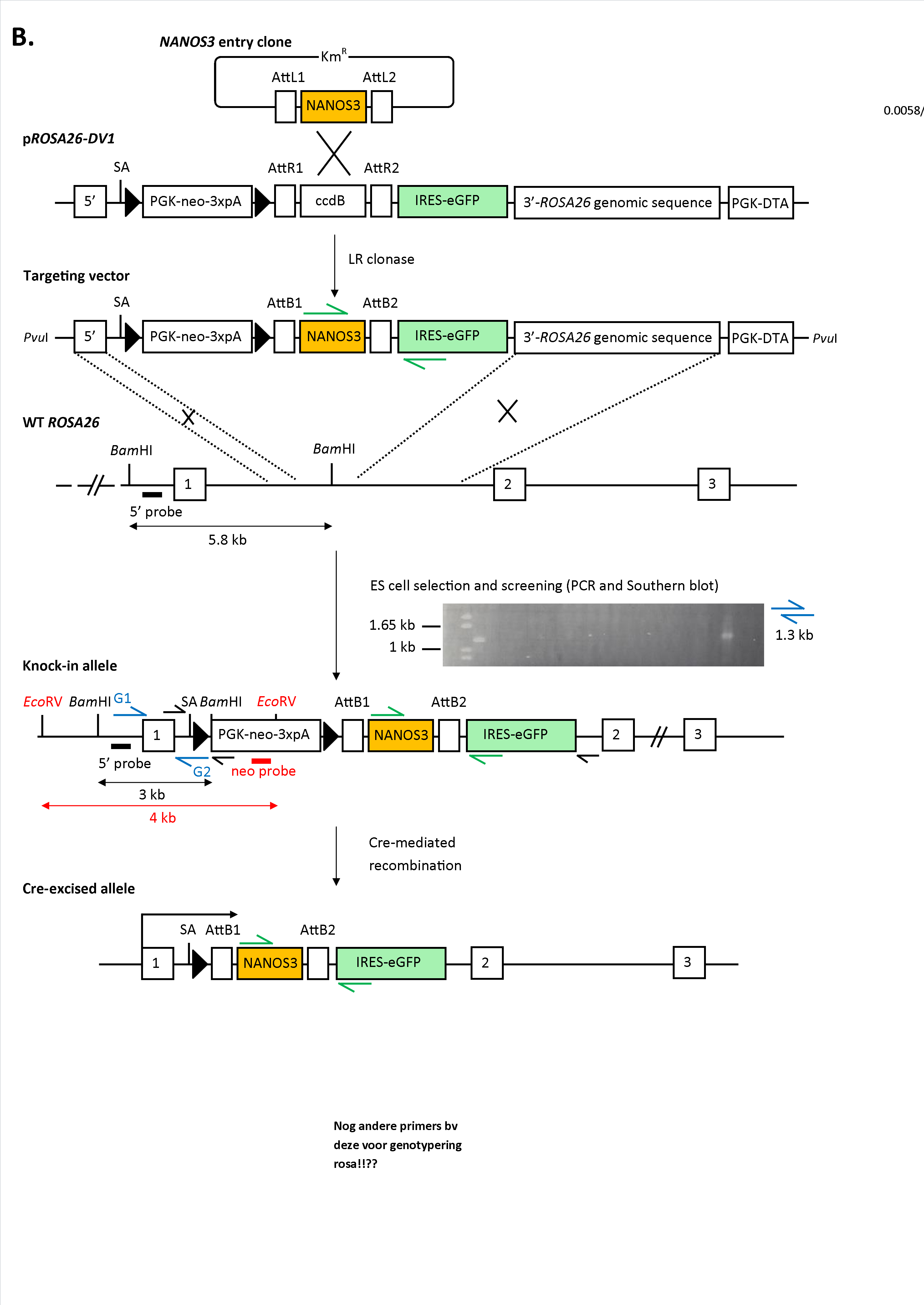


Figure S3 - Andries *et al.*

Supplement: Supplementary file 3 — Figure S3. Generation by homologous recombination into the ROSA26 locus of a transgenic mouse allowing conditional ectopic expression of a human NANOS3 allele. The Gateway® Nanos3 entry clone was recombined with the ROSA26 destination vector (pROSA26-DV1) [28], using LR clonase. The targeting vector was replicated in bacteria, subsequently linearized (PvuI) and electroporated in ES cells, where homologous recombination with the wild type (WT) ROSA26 locus took place. Correctly targeted ES cells were selected (resistance to geneticin [neomycin-resistant cells] and diphtheria toxin A [DTA]) and screened by PCR and Southern blot analyses. The blue (G1 and G2) and green (Nanos3_F and Nanos3_R) arrows represent the sequencing primers used (Fig. 1d; Additional file 20: Table S1). The black and red rectangles represent the 5′ probe and neo probe, respectively, used for Southern blot analysis (Fig. 1c). The expected band sizes after genomic DNA digestion of the WT or knock-in allele with the corresponding restriction enzymes are indicated by the double-headed arrows. Cre-mediated loxP recombination allows expression of Nanos3 and the IRES-eGFP reporter under control of the ROSA26 promoter. The resulting mice were genotyped using the primers represented by black (Rosa_F, Rosa_R1 and Rosa_R2) and green arrows (Nanos3_F and Nanos3_R) (Additional file 20: Table S1). LoxP sites are represented by triangles. SA, splice acceptor site. (DOC 374 kb) [file 12885_2019_5807_MOESM3_ESM.doc]

Nanos3<sup>LSL/-</sup>;K5Cre<sup>-/-</sup>

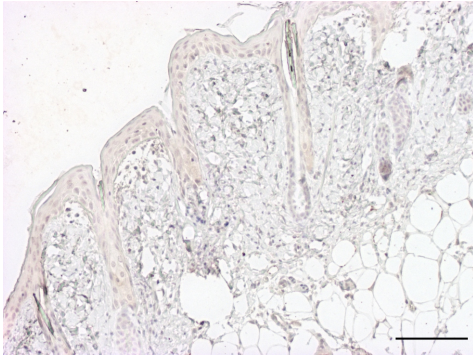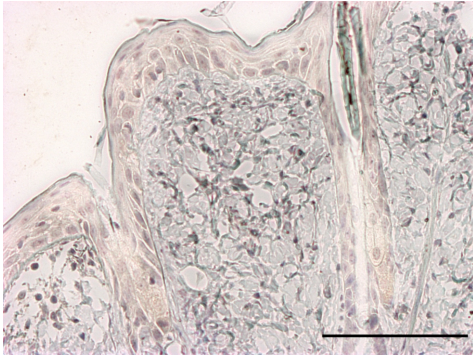

Nanos3<sup>LSL/LSL</sup>;K5Cre<sup>+/-</sup>

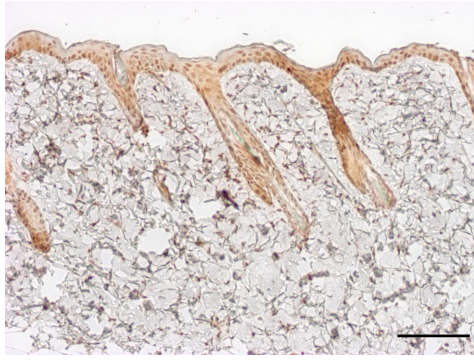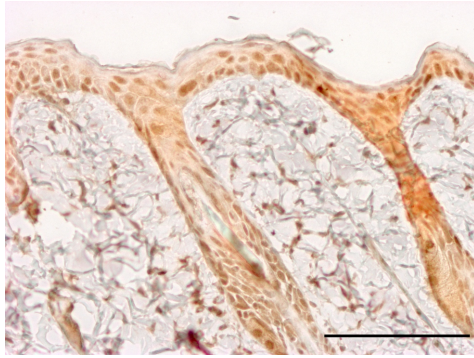

Supplement: Supplementary file 4 — Figure S4. Epidermis-specific expression of the Nanos3 transgene. eGFP expression in skin sections from a Nanos3LSL/−;K5-Cre−/− mouse and a Nanos3LSL/LSL;K5-Cre+/− mouse was analyzed by immunohistochemical staining. Bottom panels show the same fields as top panels, but with increased magnification. Bars, 100 μm. (PDF 3030 kb) [file 12885_2019_5807_MOESM4_ESM.pdf]

**Adenocarcinoma**

**Control NSCLC**

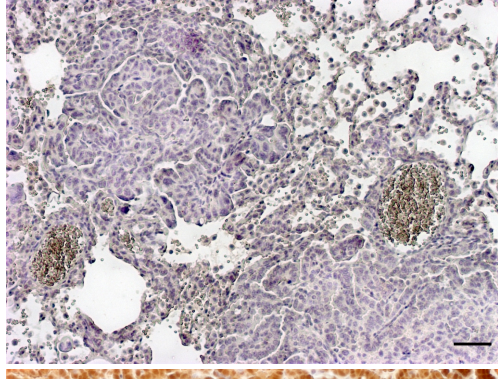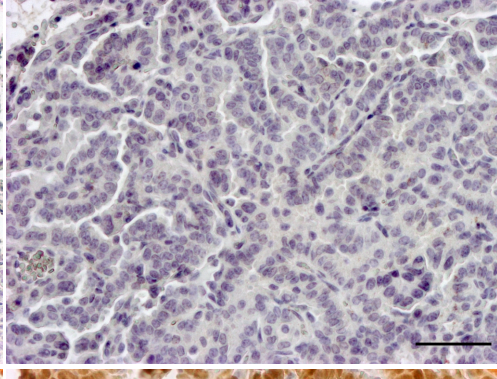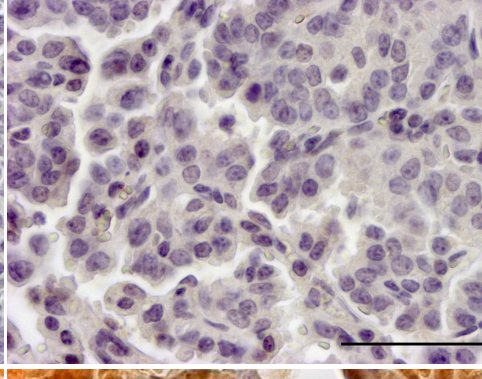

**Nanos3 NSCLC**

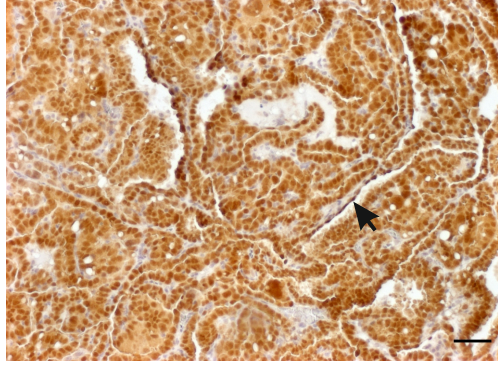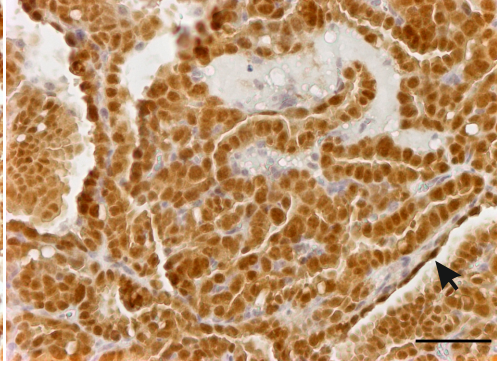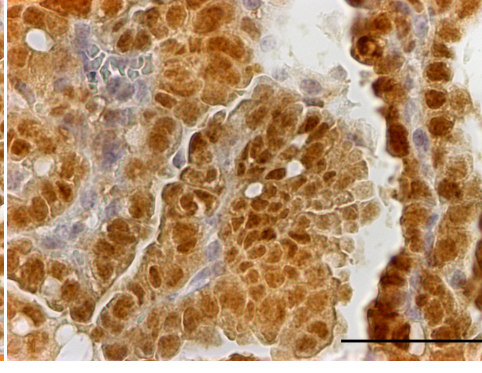

**Bronchioles**

**Control NSCLC**

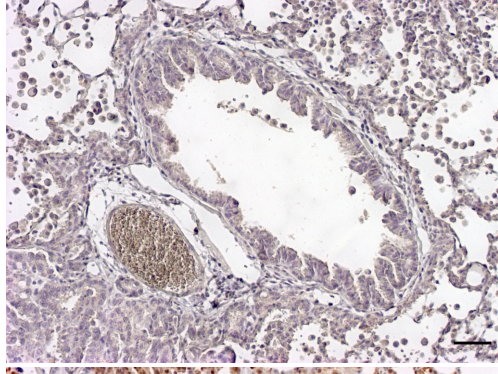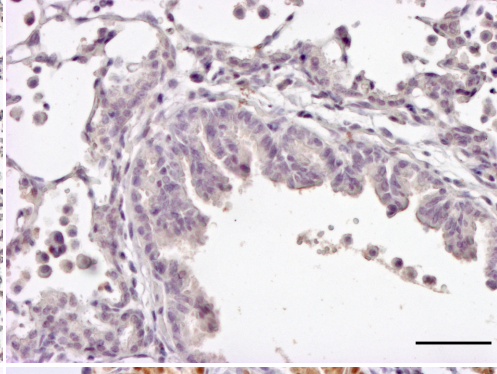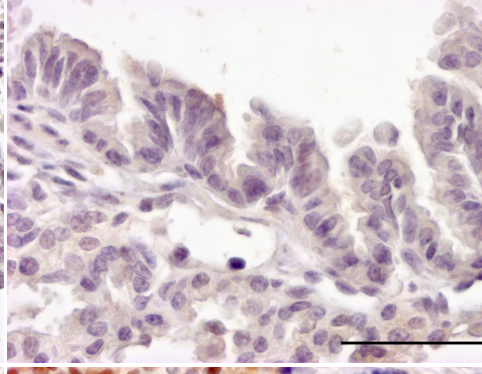

**Nanos3 NSCLC**

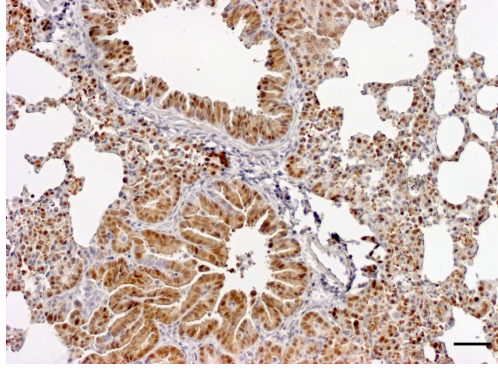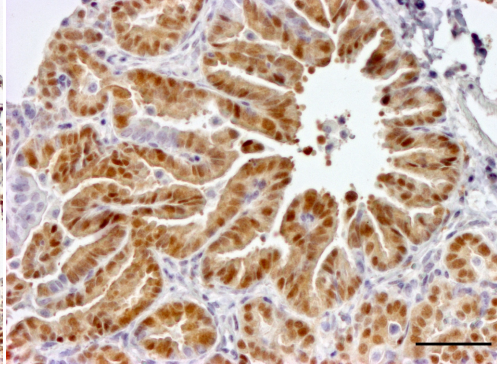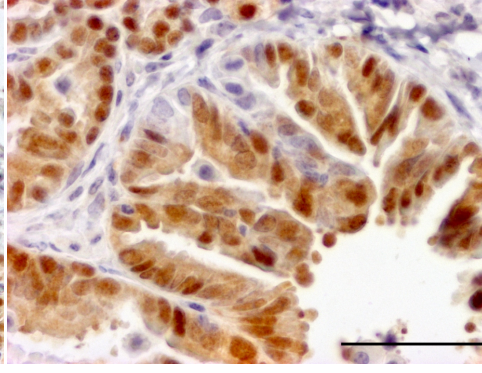

Supplement: Supplementary file 5 — Figure S5. eGFP expression in lungs of control and Nanos3 NSCLC mice. Sections of adenocarcinomas (top panels) and bronchioles (bottom panels) from control and Nanos3 NSCLC mice were stained for eGFP. Expression is evident in both alveolar and bronchiolar hyperplasia of Nanos3 NSCLC mice. Arrows point at stromal cells of an adenocarcinoma tumor. From left to right, panels correspond to images with increased magnification. Bars, 50 μm. (PDF 7230 kb) [file 12885_2019_5807_MOESM5_ESM.pdf]

**Alveolar hyperplasia**

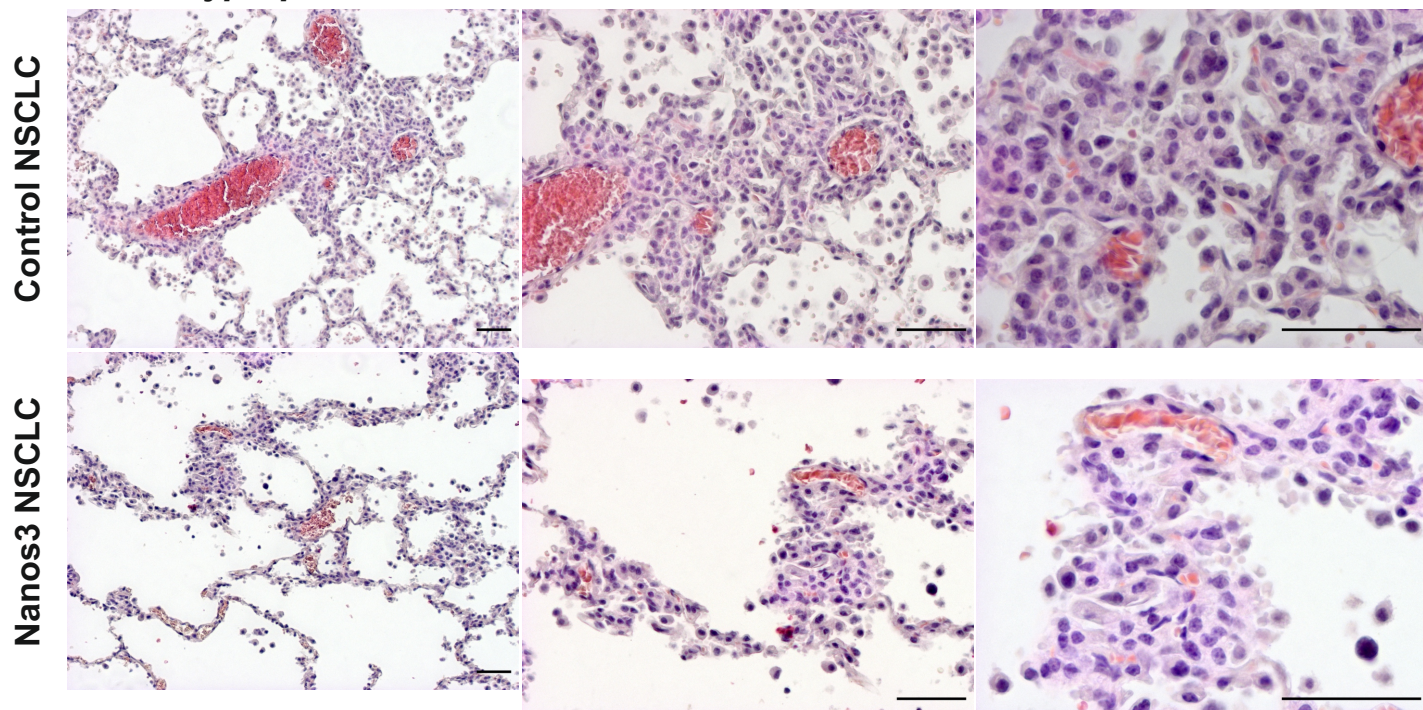

**Atypical adenomatous hyperplasia**

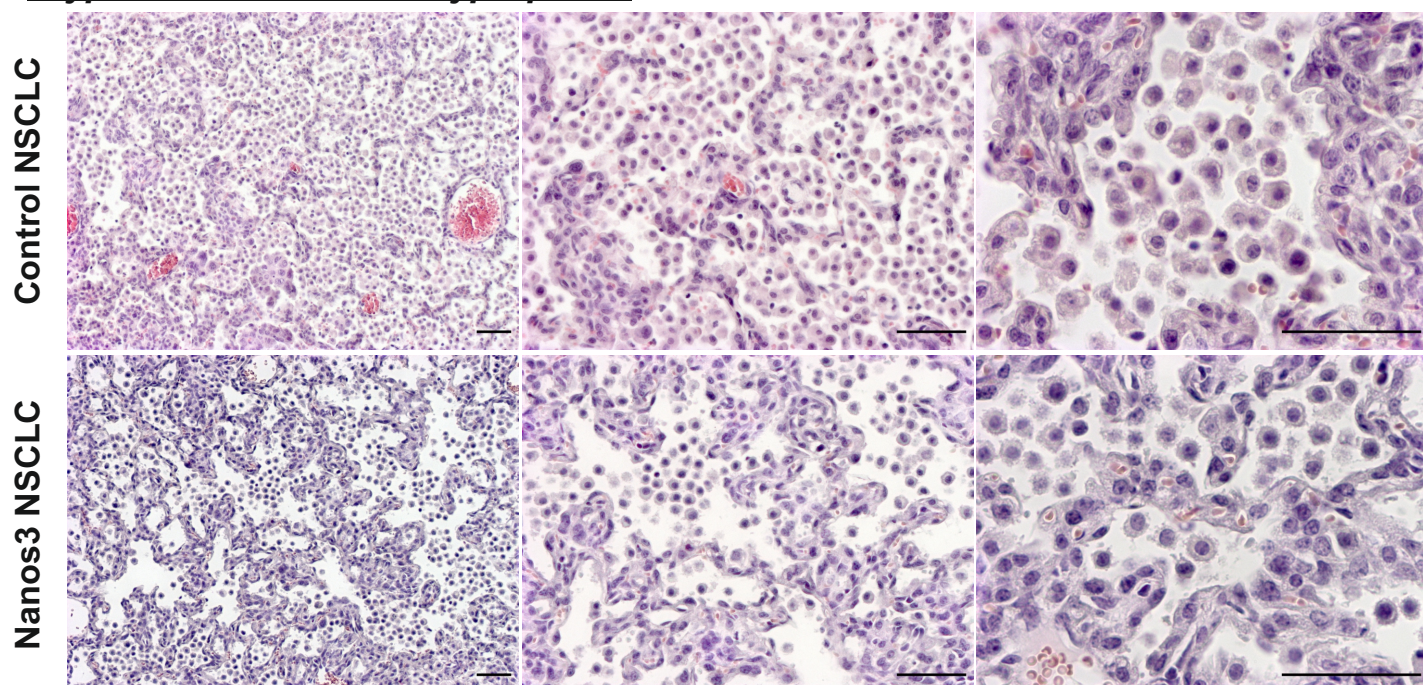

**Adenocarcinoma**

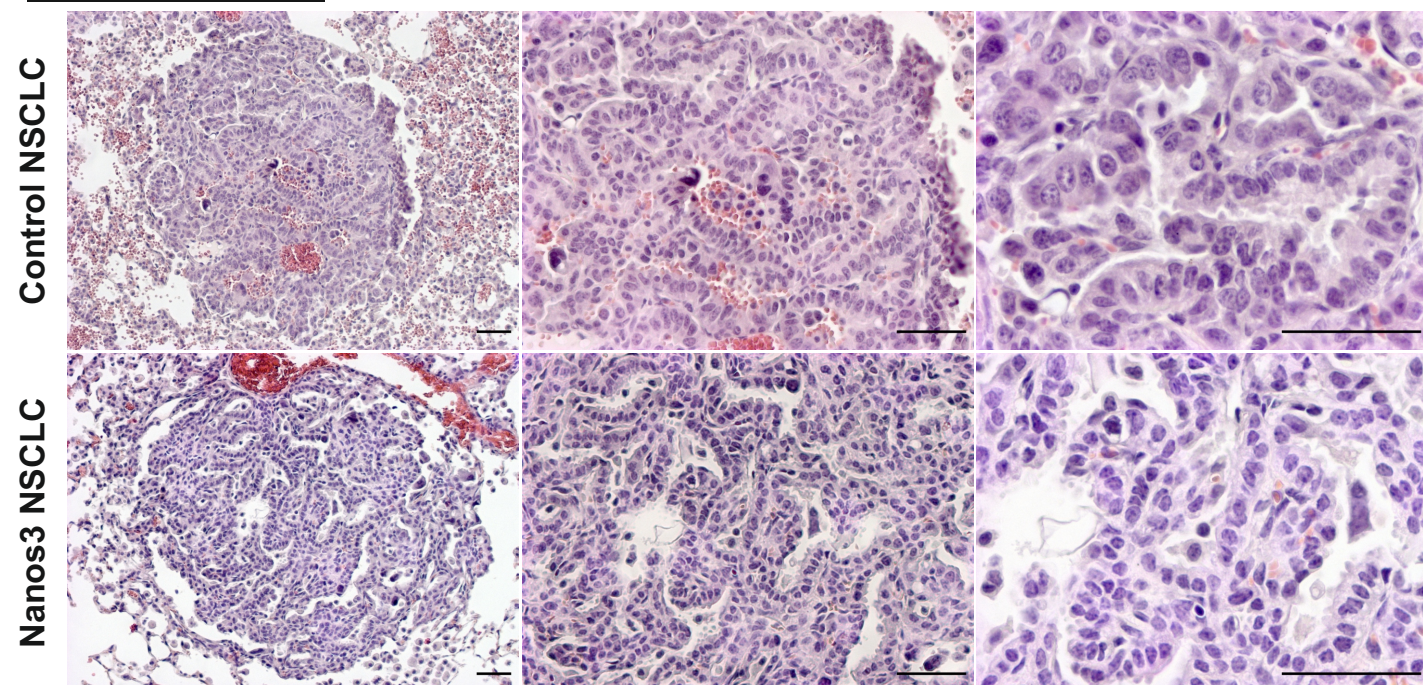

Supplement: Supplementary file 6 — Figure S6. Microscopic images of H&E-stained lung sections from control and Nanos3 NSCLC mice show different stages of tumor progression in the alveolar spaces. Alveolar hyperplasia, premalignant atypical adenomatous hyperplasia and adenocarcinoma were observed in the alveolar spaces of both control and Nanos3 NSCLC mice. Panels correspond to increasing magnification from left to right. Bars, 50 μm. (PDF 8592 kb) [file 12885_2019_5807_MOESM6_ESM.pdf]

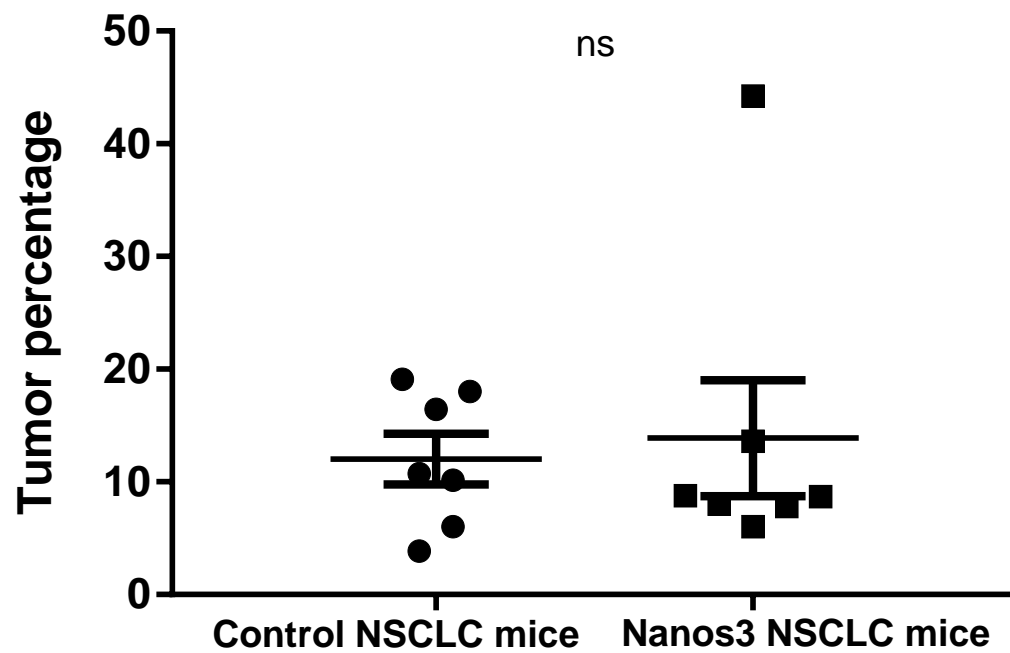

Supplement: Supplementary file 8 — Figure S8. The tumor percentage of the lungs is comparable in control and Nanos3 NSCLC mice. Five H&E sections throughout the complete lungs were used to measure the tumor mass by scanning followed by appropriate image analysis as detailed in Methods. Quantification was done with ImageJ. Error bars, SEM. (PDF 9 kb) [file 12885_2019_5807_MOESM8_ESM.pdf]

**Bronchioles**

**Nanos3 NSCLC**

**Control NSCLC**

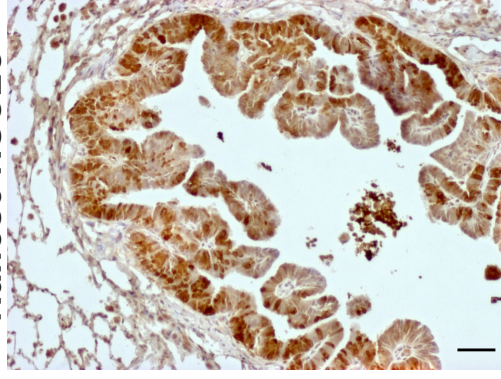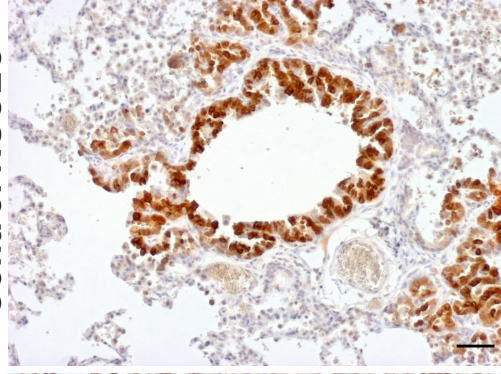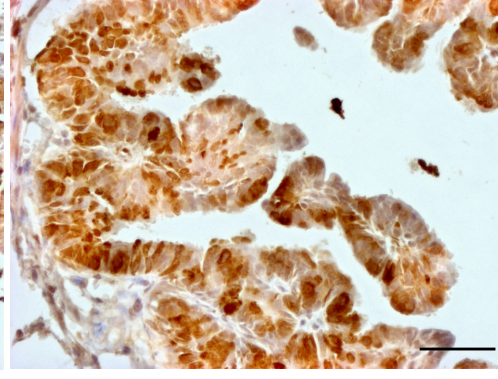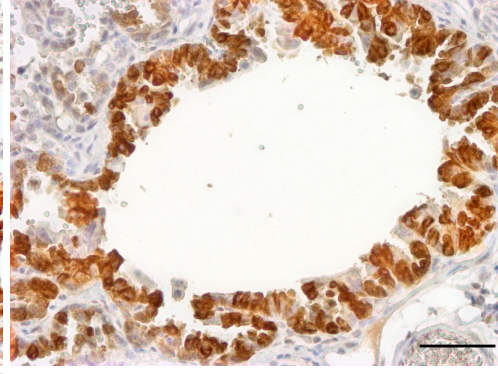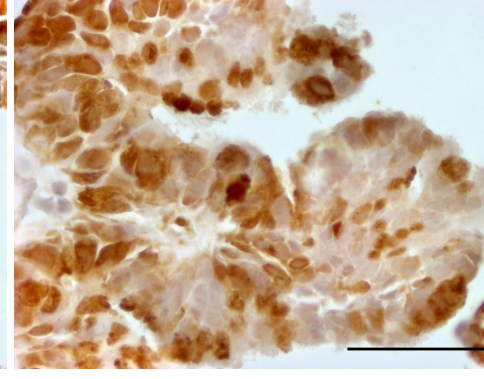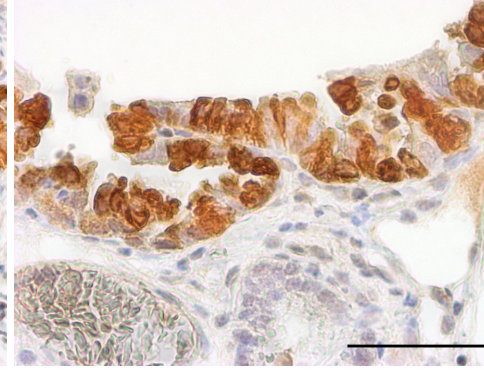

**Adenocarcinoma**

**Nanos3 NSCLC**

**Control NSCLC**

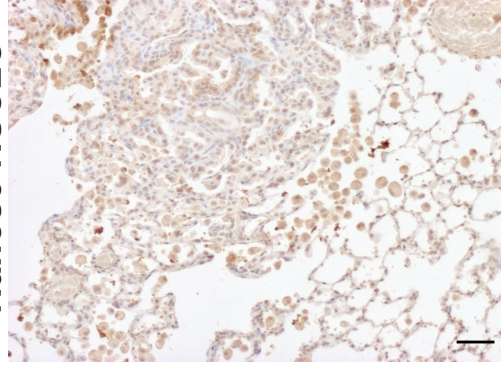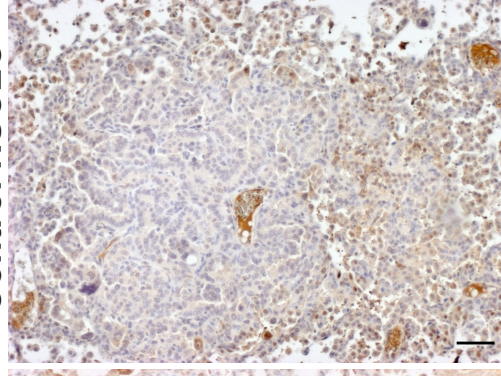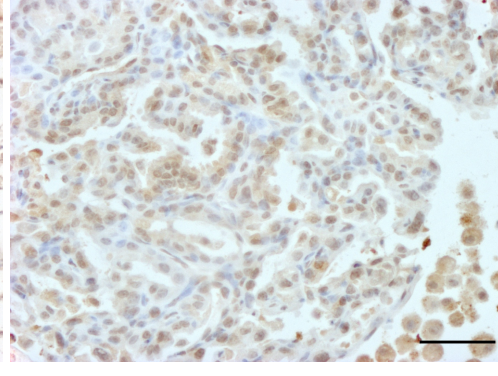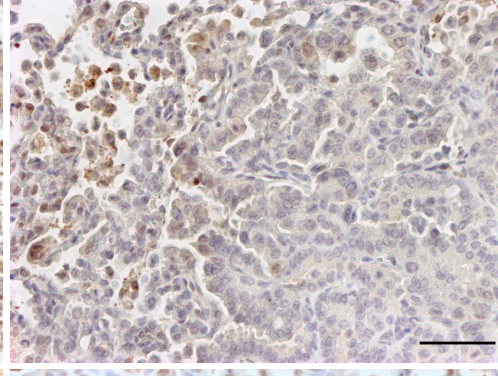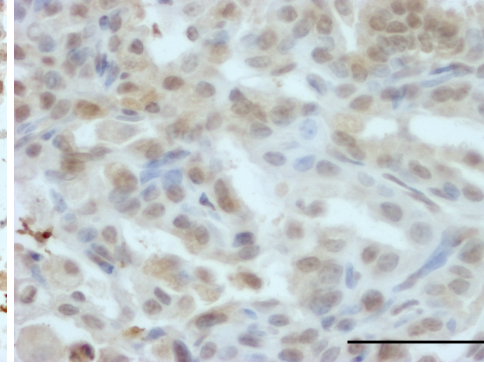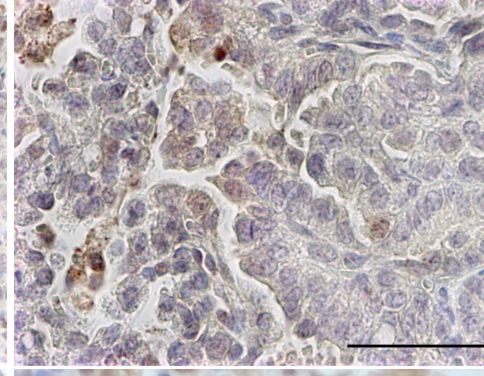

Supplement: Supplementary file 9 — Figure S9. CC10 expression in adenocarcinomas and bronchioles of control and Nanos3 NSCLC mice. CC10 staining of lung sections of adenocarcinomas (top panels) and bronchioles (bottom panels) from control and Nanos3 NSCLC mice. Panels correspond to increasing magnification from left to right. Bars, 50 μm. (PDF 6460 kb) [file 12885_2019_5807_MOESM9_ESM.pdf]

**Bronchioles**

**Nanos3 NSCLC**

**Control NSCLC**

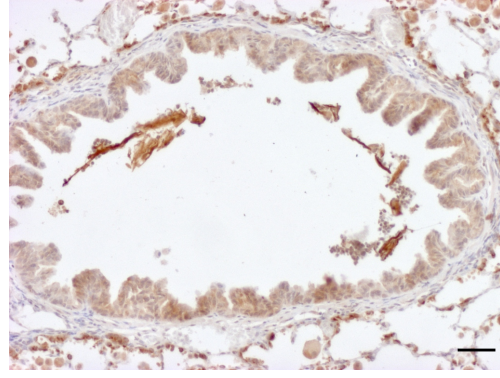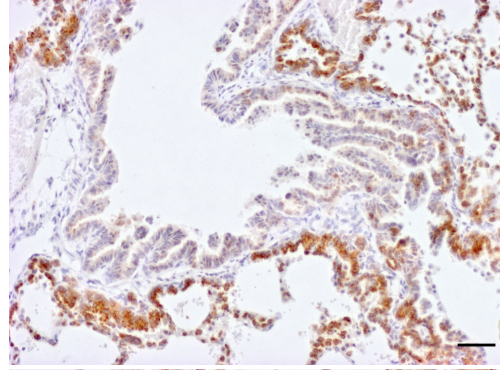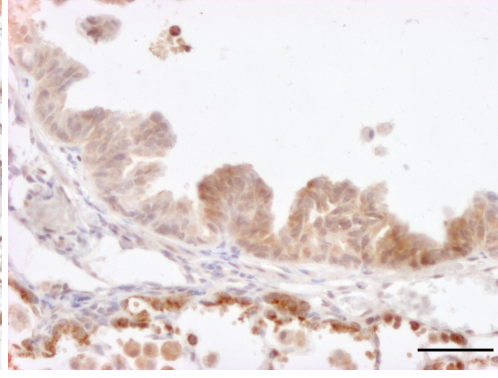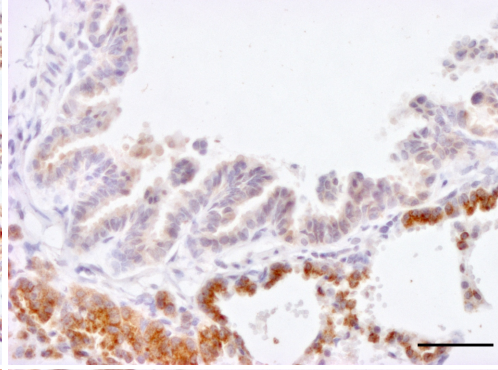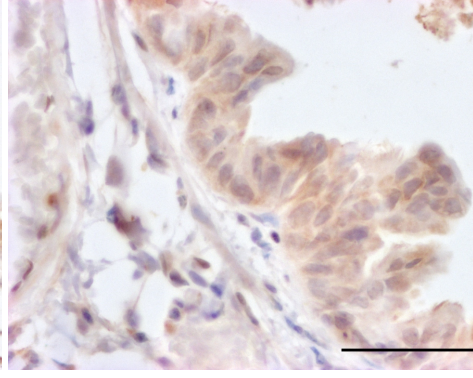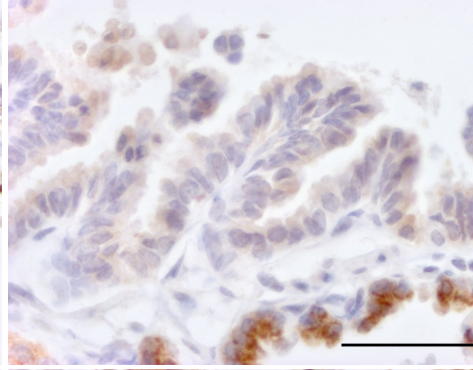

**Adenocarcinoma**

**Nanos3 NSCLC**

**Control NSCLC**

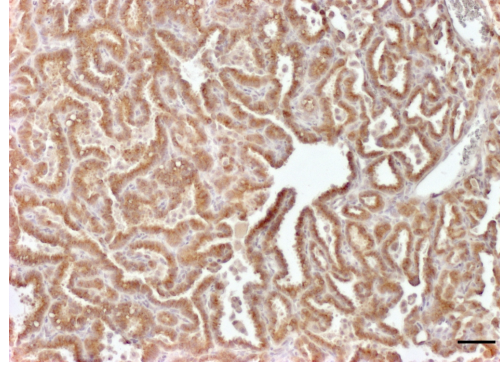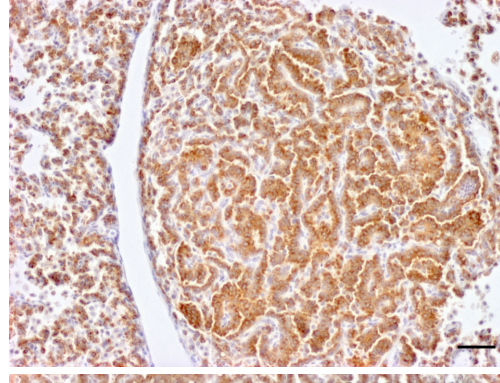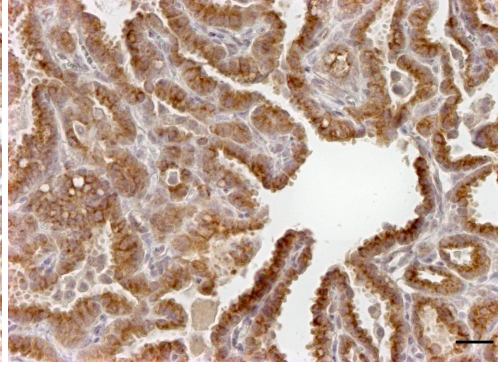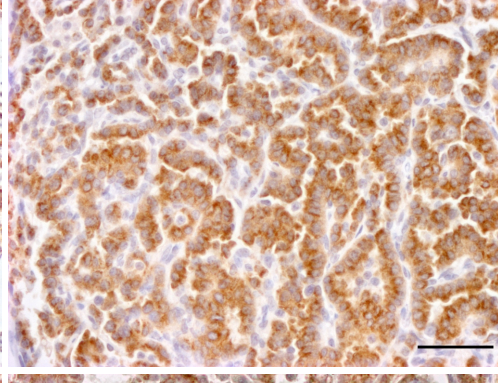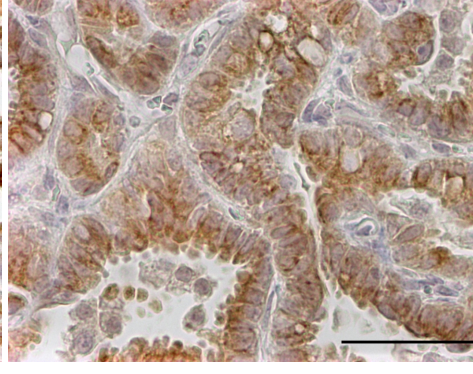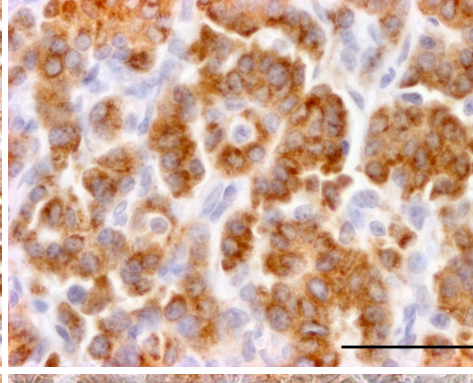

Supplement: Supplementary file 10 — Figure S10. SPC expression in adenocarcinomas and bronchioles of control and Nanos3 NSCLC mice. SPC staining of lung sections of adenocarcinomas (top panels) and bronchioles (bottom panels) from control and Nanos3 NSCLC mice. Panels correspond to increasing magnification from left to right. Bars, 50 μm. (PDF 6333 kb) [file 12885_2019_5807_MOESM10_ESM.pdf]

**Bronchioles**

**Nanos3 NSCLC**

**Control NSCLC**

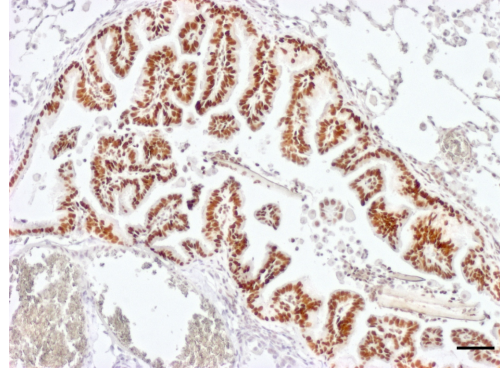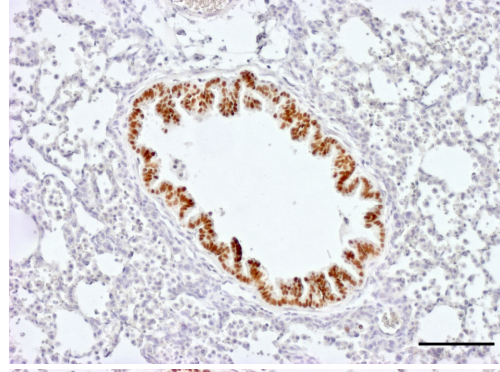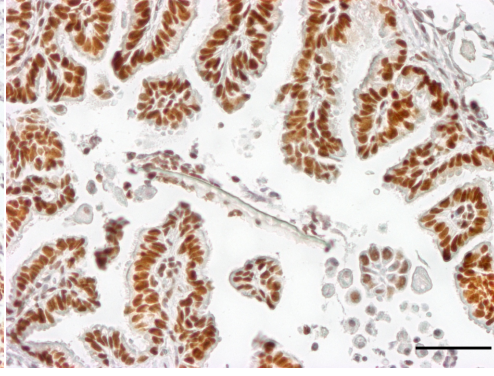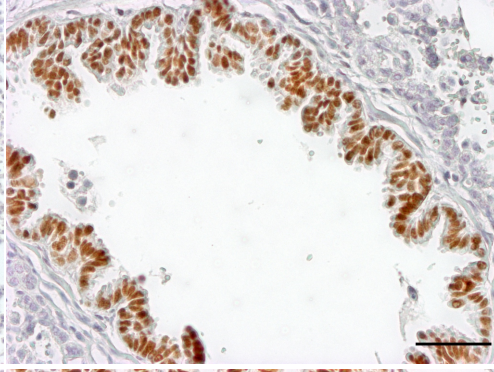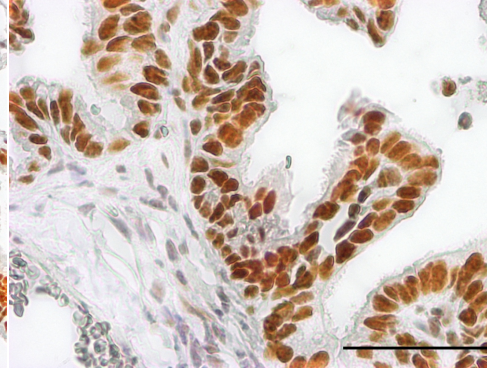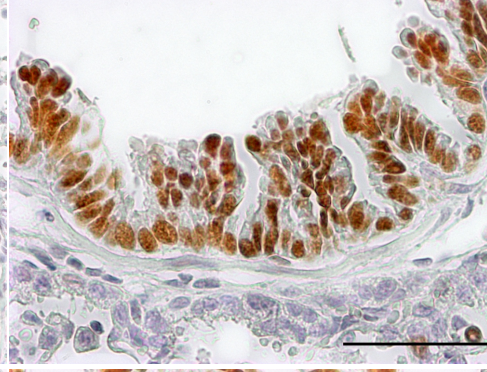

**Adenocarcinoma**

**Nanos3 NSCLC**

**Control NSCLC**

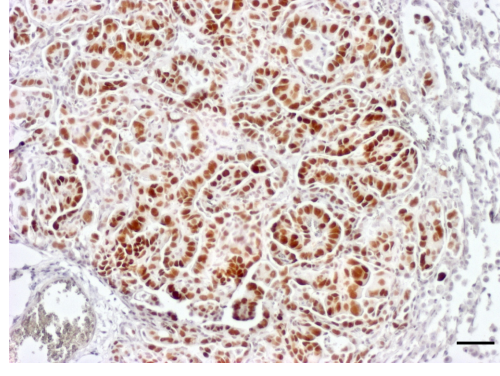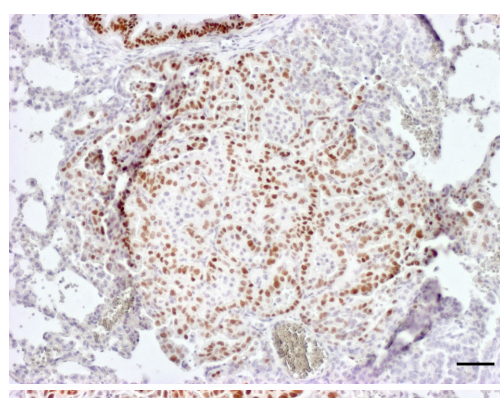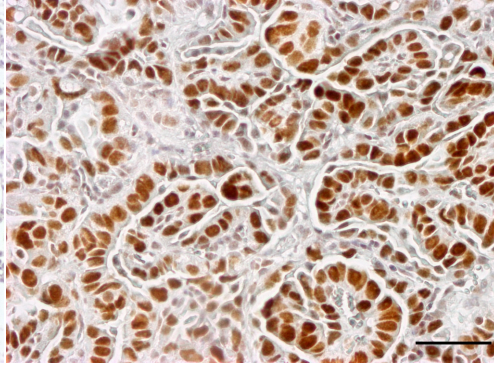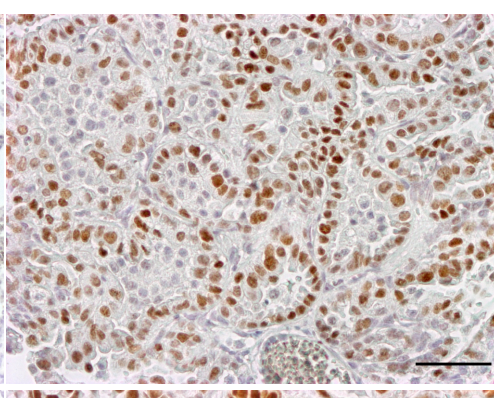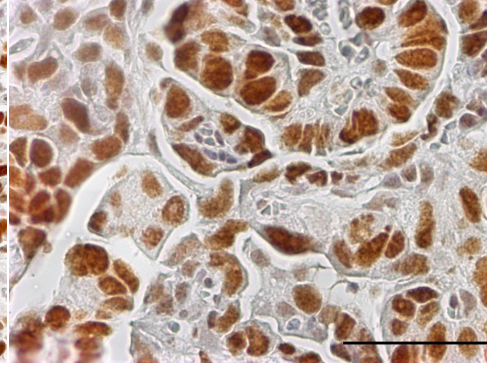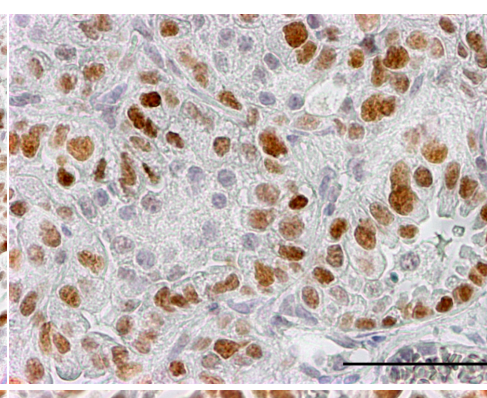

Supplement: Supplementary file 11 — Figure S11. Sox2 expression in adenocarcinomas and bronchioles of control and Nanos3 NSCLC mice. Sox2 staining of lung sections of adenocarcinomas (top panels) and bronchioles (bottom panels) from control and Nanos3 NSCLC mice. Panels correspond to increasing magnification from left to right. Bars, 50 μm. (PDF 6975 kb) [file 12885_2019_5807_MOESM11_ESM.pdf]

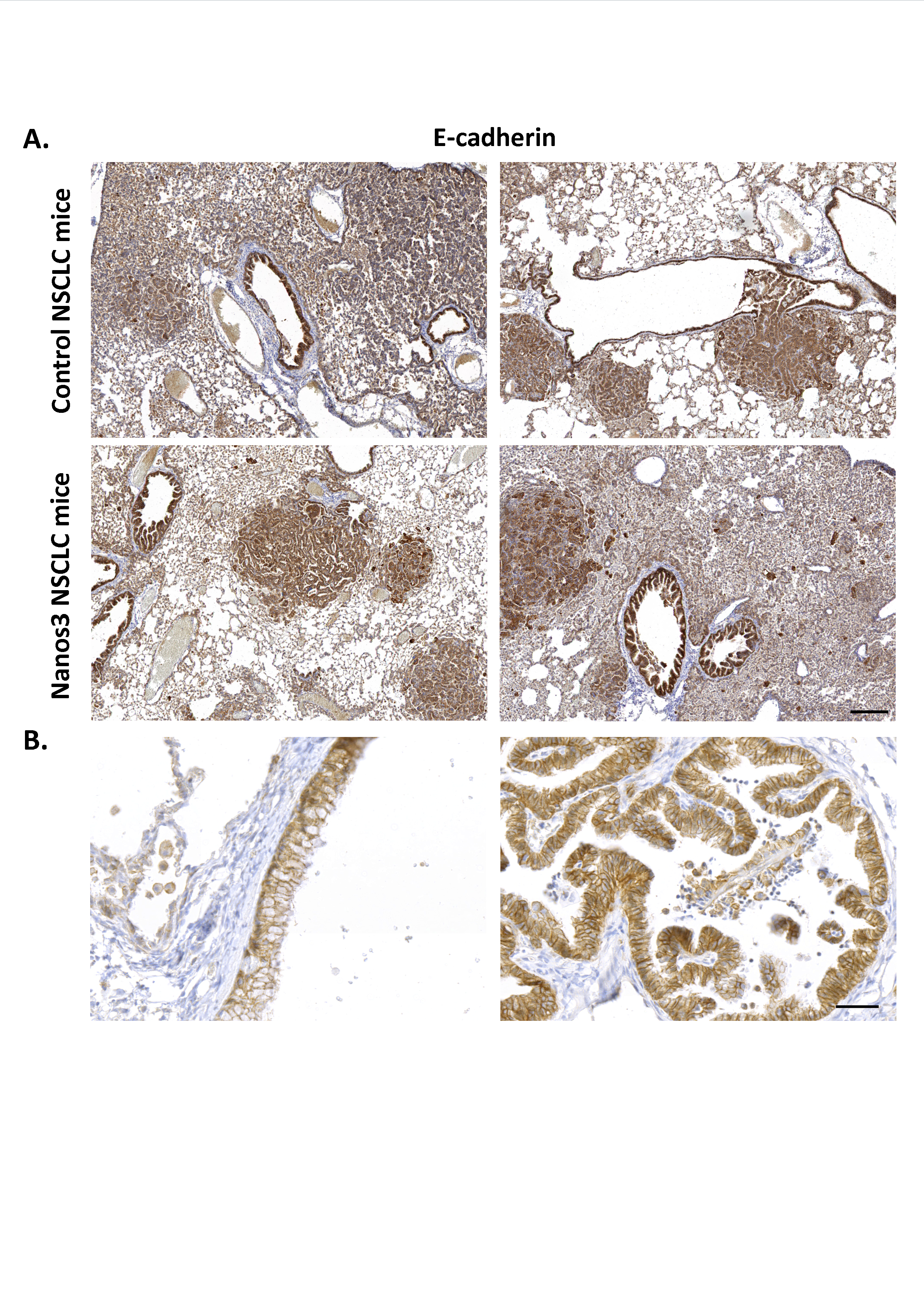


Figure S12 - Andries *et al.*

Supplement: Supplementary file 12 — Figure S12. E-cadherin expression in the bronchioles and adenocarcinomas of NSCLC mice. A. E-cadherin staining of lung sections from control (LSL-KRasG12D;p53fl/fl;CCSP-rtTA+/−;TetO-Cre+/−) and Nanos3 (Nanos3LSL/−;LSL-KRasG12D;p53fl/fl;CCSP-rtTA+/−;TetO-Cre+/−) NSCLC mice. Bar, 200 μm. B. Both normal and hyperplastic bronchioles stained positive for E-cadherin. Bar, 50 μm. (DOC 12361 kb) [file 12885_2019_5807_MOESM12_ESM.doc]

**Bronchioles**

**Nanos3 NSCLC**

**Control NSCLC**

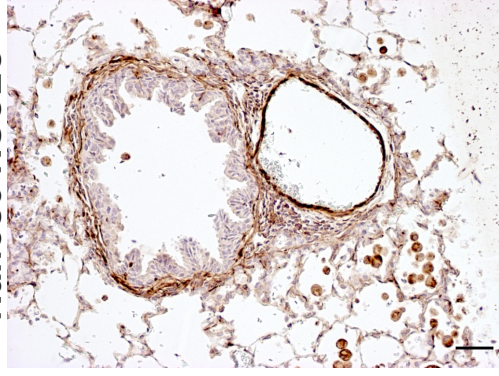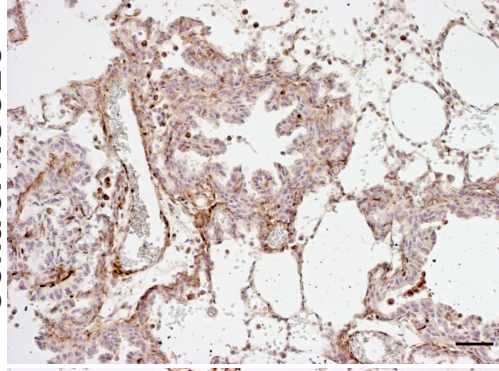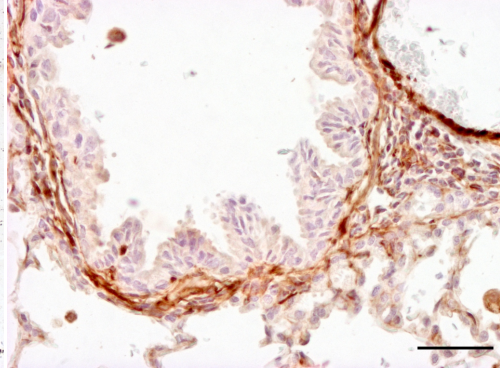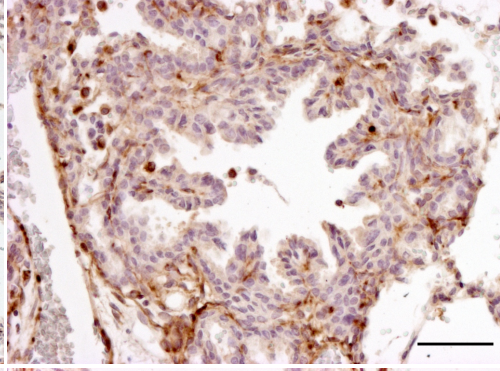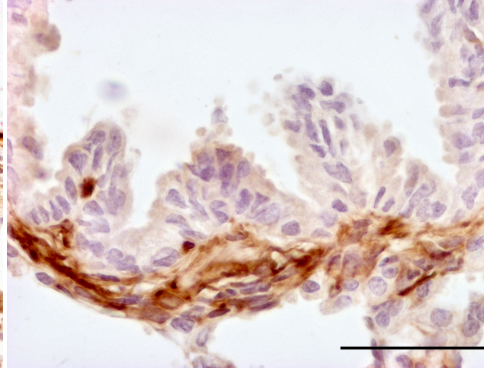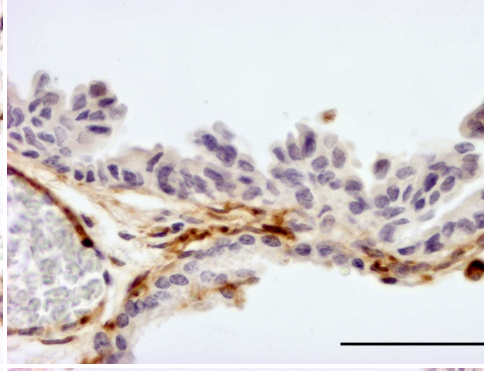

**Adenocarcinoma**

**Nanos3 NSCLC**

**Control NSCLC**

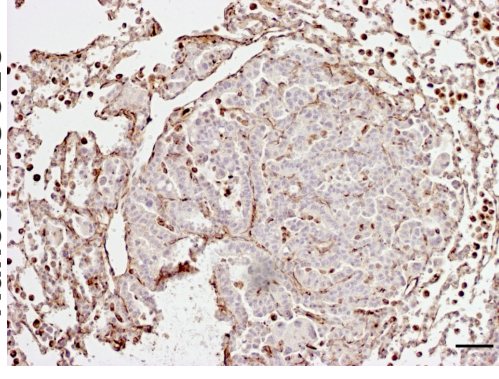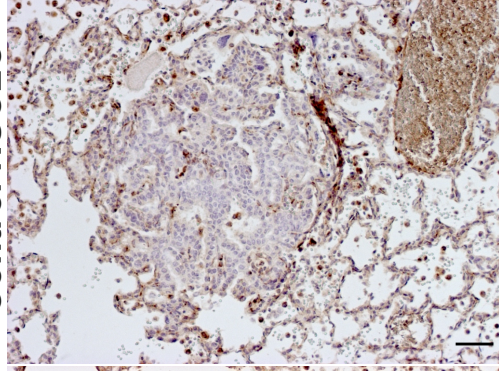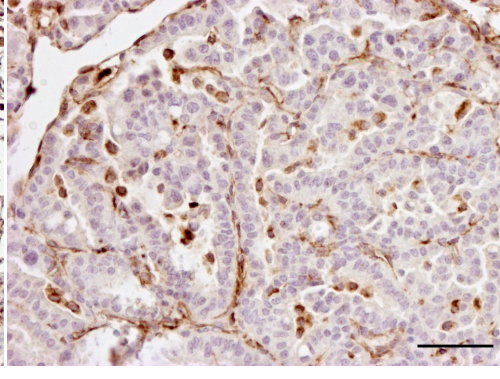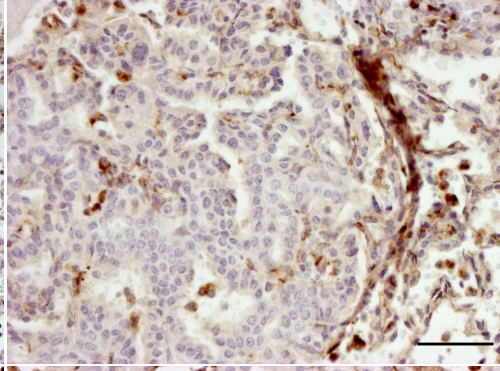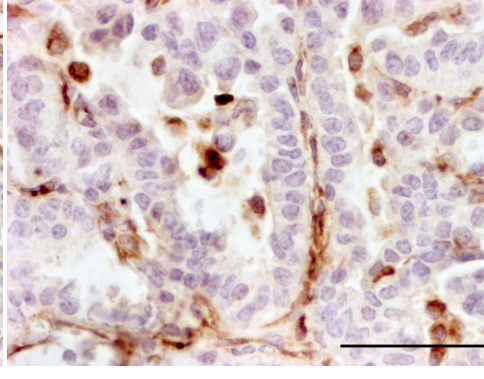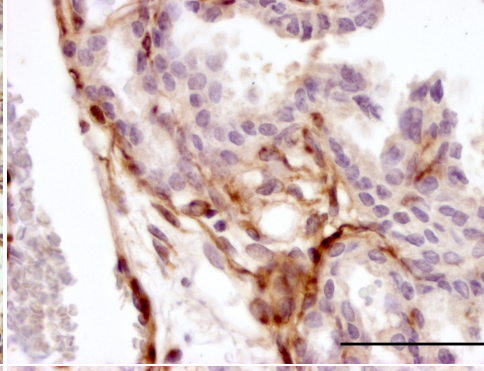

Supplement: Supplementary file 13 — Figure S13. Vimentin expression in adenocarcinomas and bronchioles of control and Nanos3 NSCLC mice. Vimentin staining of lung sections of adenocarcinomas (top panels) and bronchioles (bottom panels) from control and Nanos3 NSCLC mice showed similar vimentin expression patterns for control and Nanos3 NSCLC mice. Panels correspond to increasing magnification from left to right. Bars, 50 μm. (PDF 6633 kb) [file 12885_2019_5807_MOESM13_ESM.pdf]

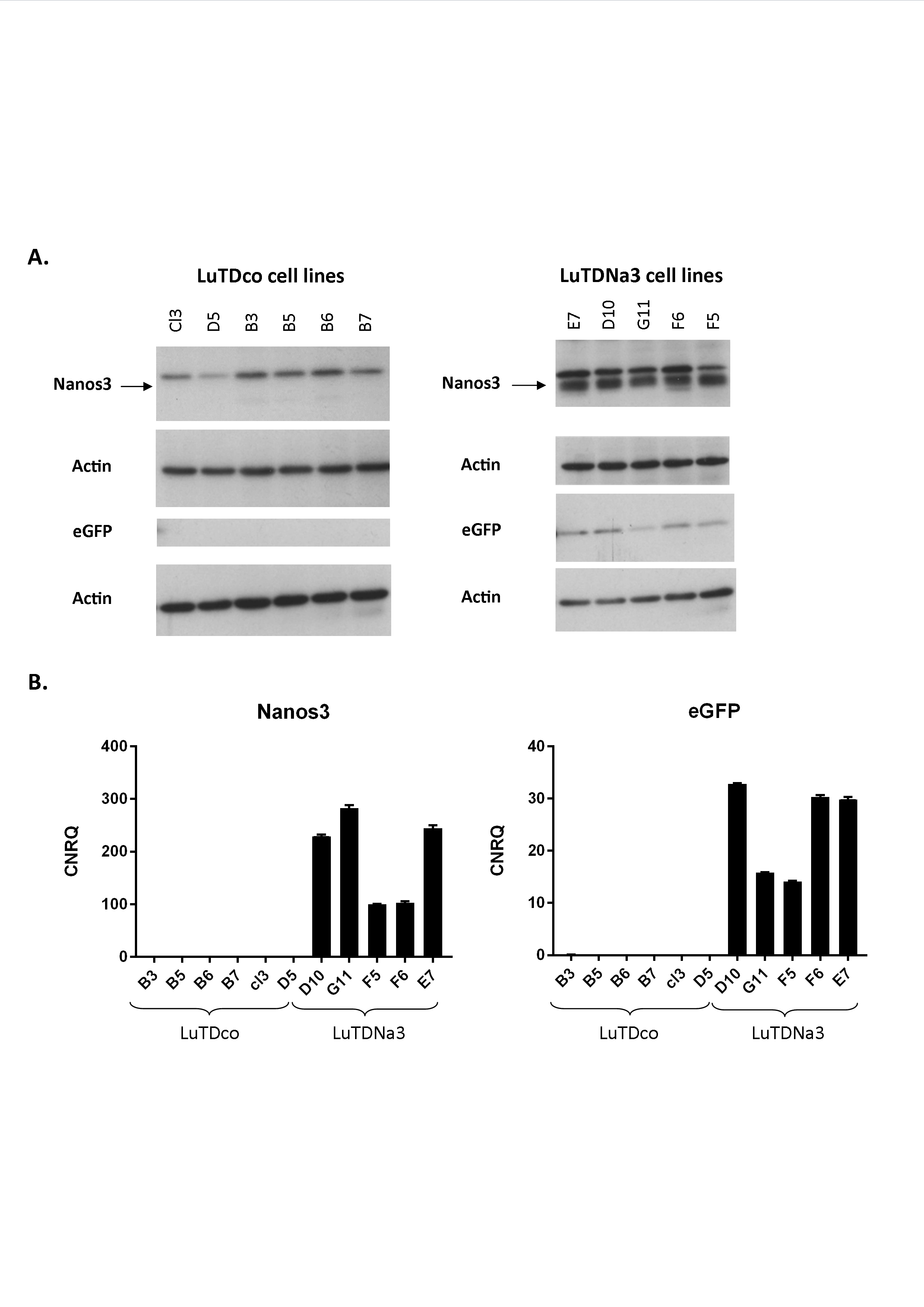


Figure S14 - Andries *et al.*

Supplement: Supplementary file 14 — Figure S14. Nanos3 and eGFP expression of primary lung cancer cell cultures. Primary cell cultures derived from the lungs of a control NSCLC mouse (LuTDco) and a Nanos3 overexpressing NSCLC mouse (LuTDNa3) were tested for Nanos3 and eGFP expression by western blotting (A) and RT-qPCR (B). Actin was used as a loading control for western blot analysis. CNRQ, calibrated normalized relative quantity, error bars, SEM; n = 3. Gene expression was normalized to reference genes (rpl13A, ywhaz and sdha) using qbase+ (Biogazelle) [35]. (DOC 654 kb) [file 12885_2019_5807_MOESM14_ESM.doc]

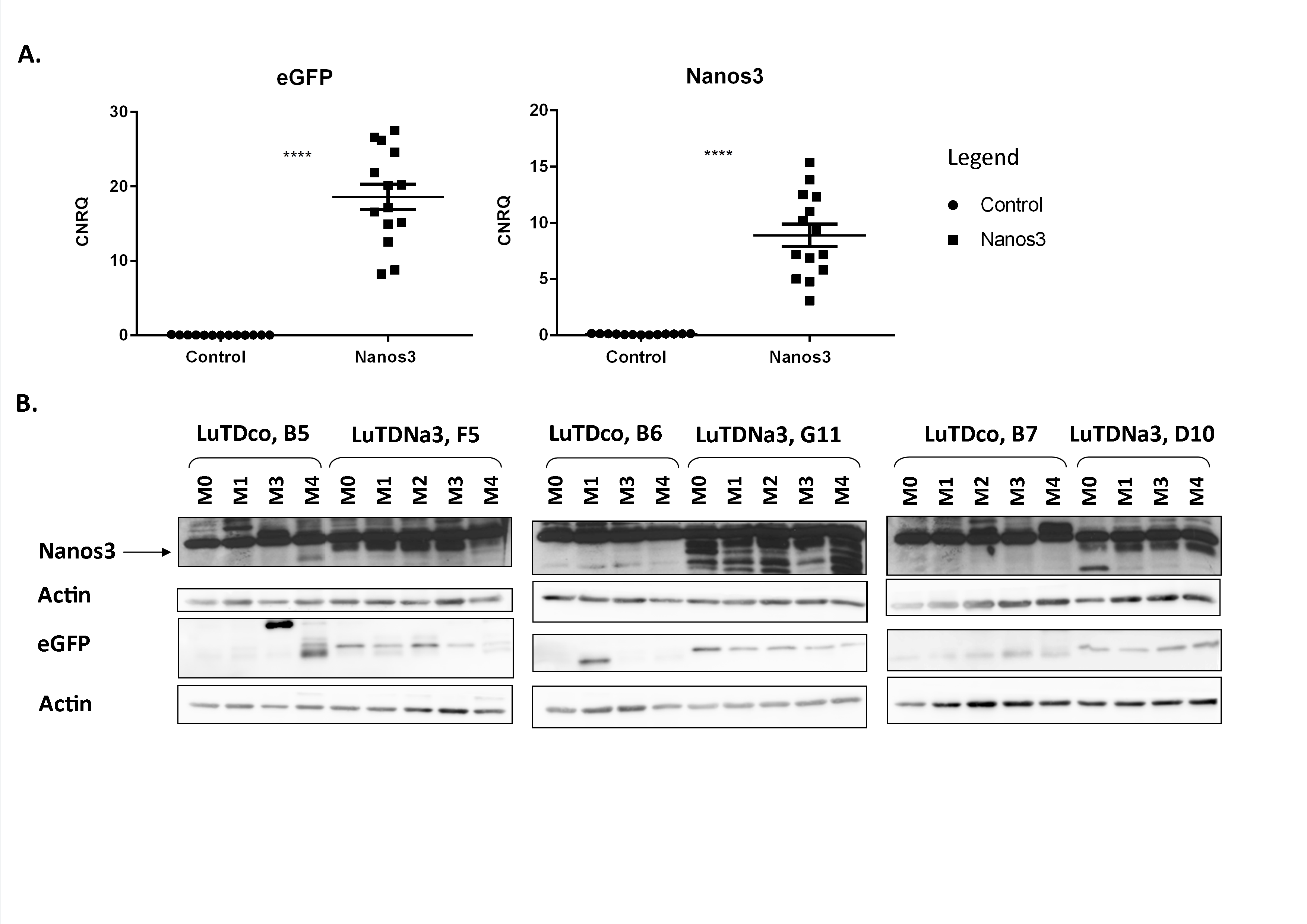


Figure S15 - Andries *et al.*

Supplement: Supplementary file 15 — Figure S15. Analysis of eGFP and NANOS3 mRNA and protein expression in ectopic tumors (allografts) from control and Nanos3-expressing lung tumor-derived cell cultures. Ectopic tumors were dissected from athymic mice injected subcutaneously with cultured primary lung cancer cells derived from either a LSL-KRasG12D;p53fl/fl; CCSP-rtTA+/−;TetO-Cre+/− mouse (LuTDco) or a Nanos3LSL/−;LSL-KRasG12D;p53fl/fl;CCSP-rtTA+/−;TetO-Cre+/− mouse (LuTDNa3). A. RNA lysates were made from part of the ectopic subcutaneous tumors originating from LuTDco or LuTDNa3 cell cultures. Each dot represents an ectopic tumor from an athymic mouse injected with these cell cultures. CNRQ, calibrated normalized relative quantity. Error bars, SEM; ns: not significant, ****: P ≤ 0.0001. Gene expression was normalized to reference genes (rpl13A, ywhaz and sdha) using qbase+ (Biogazelle) [35]. B. Protein lysates from the allografts from the injected mice (M0 to M4) were checked for Nanos3 and eGFP expression by western blotting. Actin was used as a loading control. (DOC 768 kb) [file 12885_2019_5807_MOESM15_ESM.doc]

**A.**

LuTDco

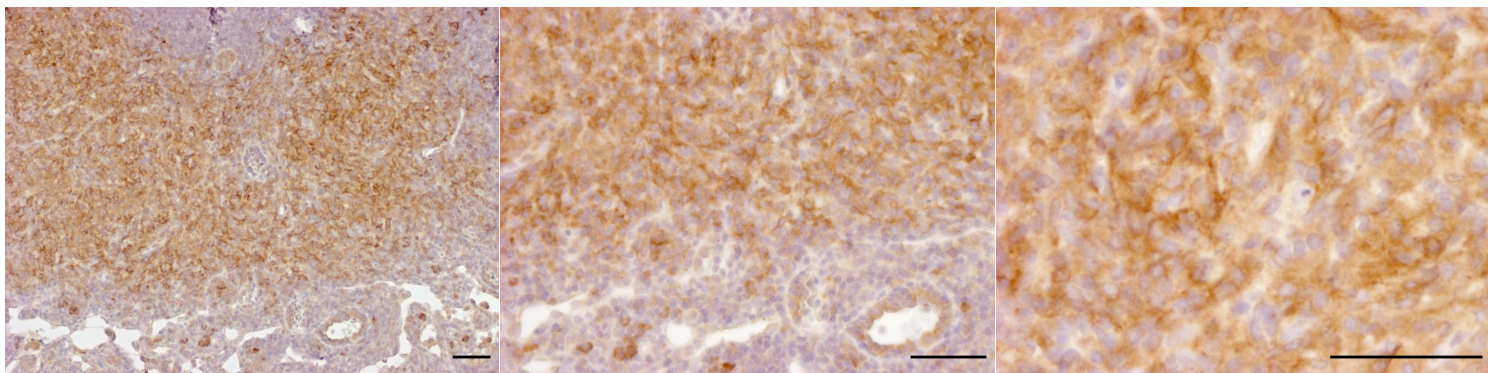

LuTDNa3

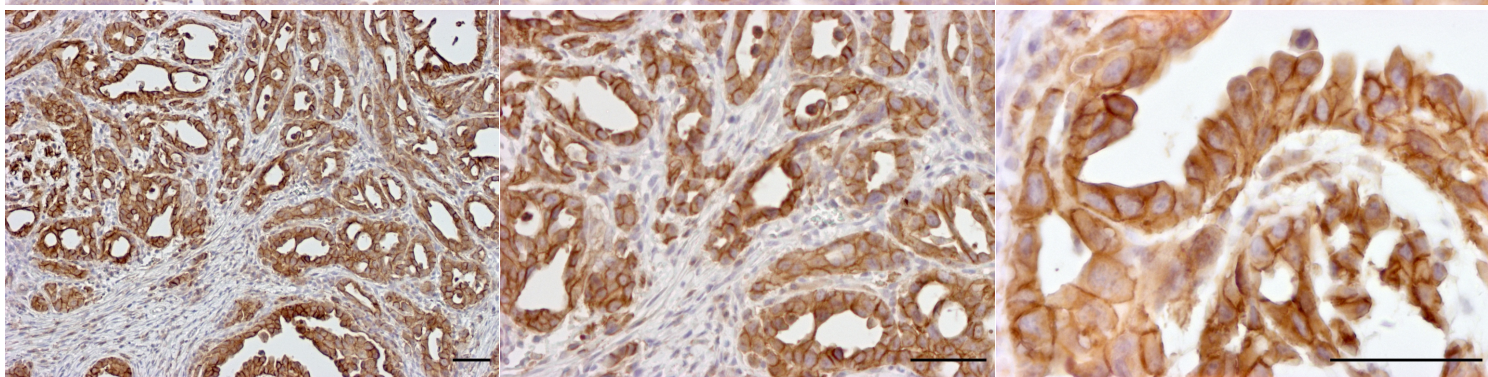

**B.**

LuTDco

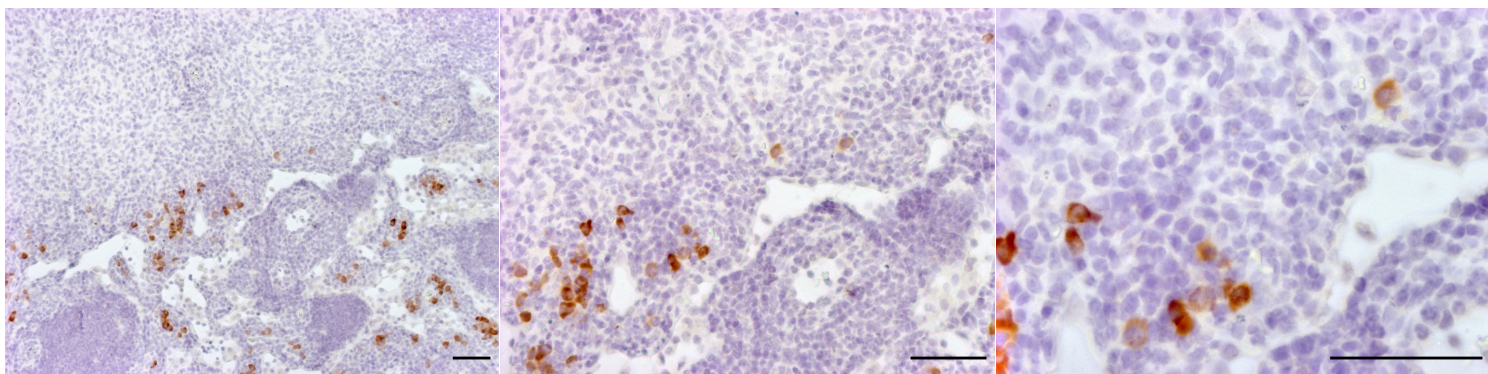

LuTDNa3

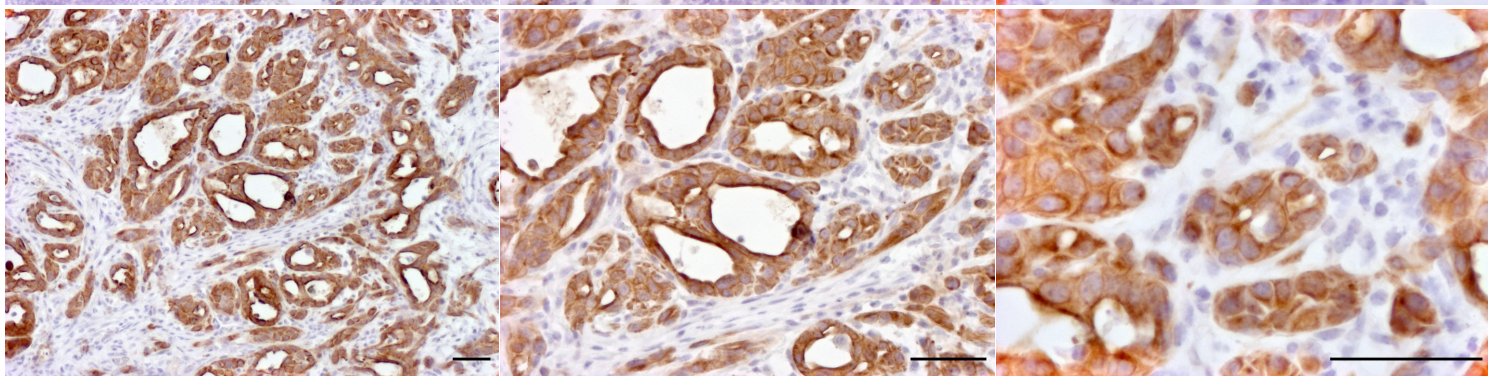

Supplement: Supplementary file 16 — Figure S16. Epithelial differentiation in lymph node metastasis of mice subcutaneously injected with LuTDNa3 cell cultures. Lymph node sections were stained for E-cadherin (A) and pan-cytokeratin (B), providing proof for the epithelial origin and the obvious differentiation of the lymph node metastases. Panels correspond to increasing magnification from left to right. Bars, 50 μm. (PDF 6514 kb) [file 12885_2019_5807_MOESM16_ESM.pdf]

**A.**

LuTDco

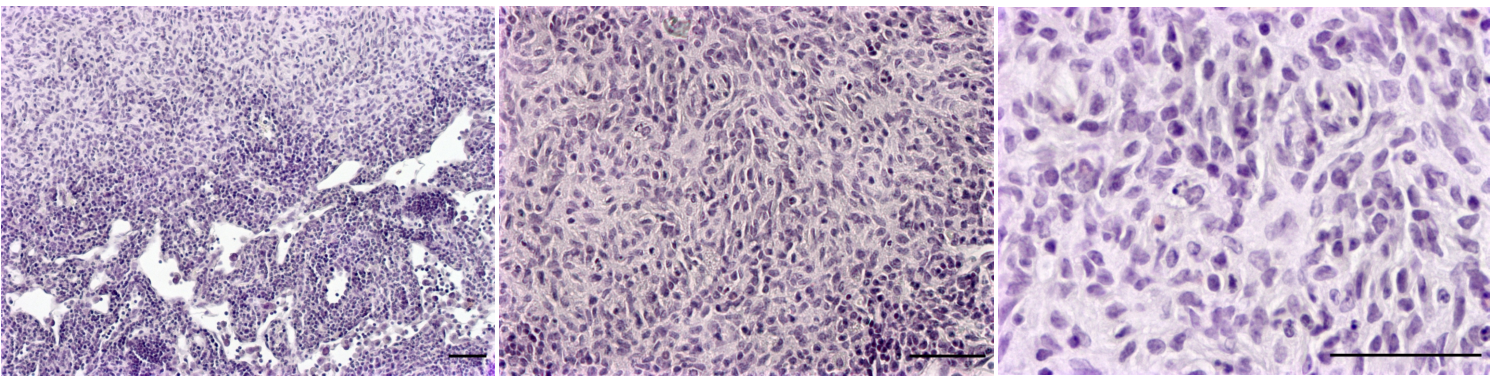

LuTDNa3

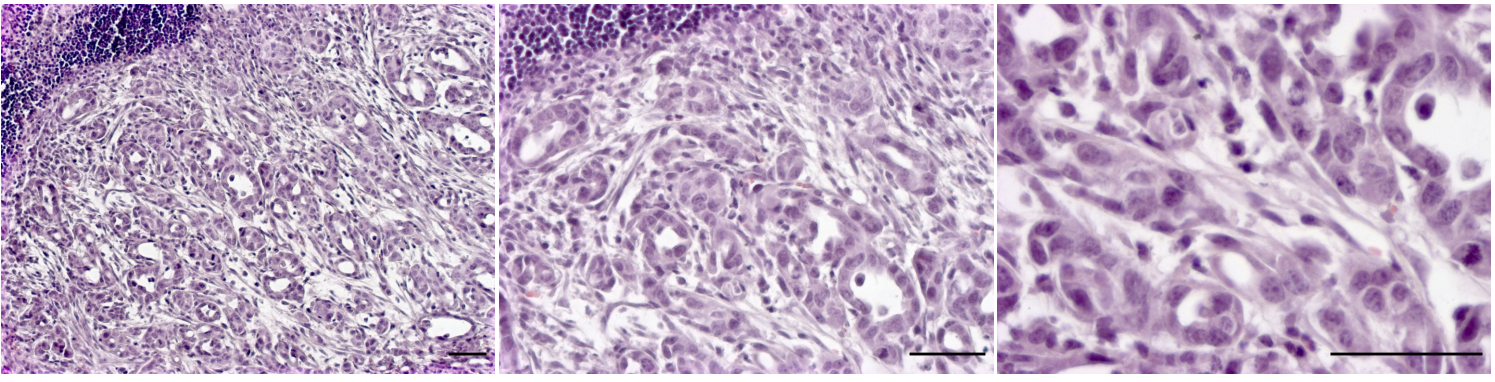

**B.**

LuTDco

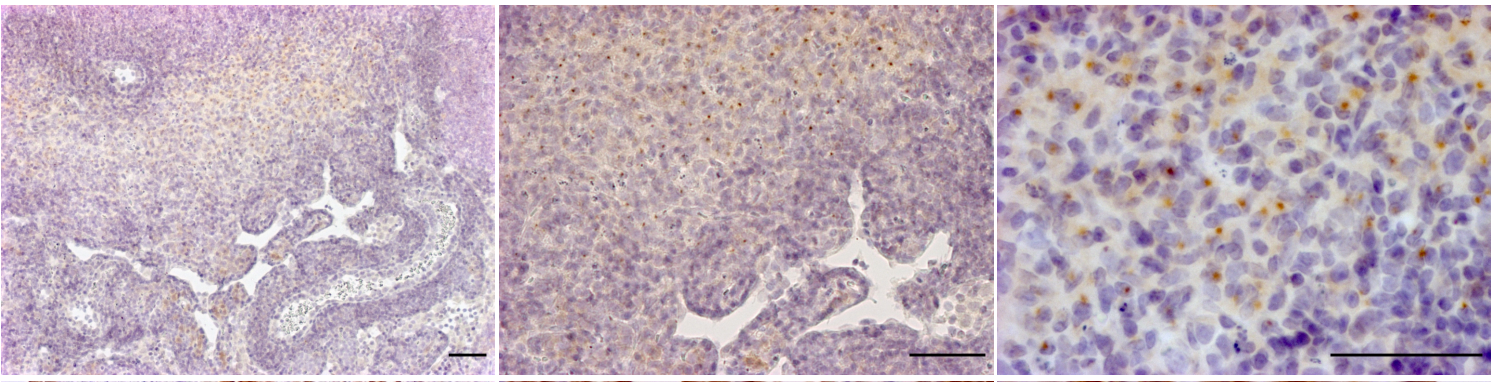

LuTDNa3

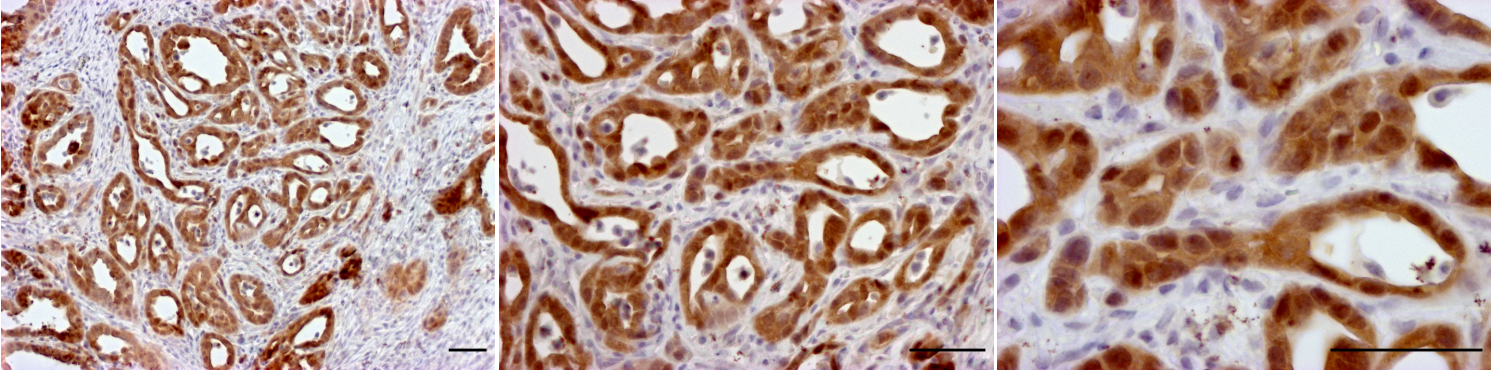

Supplement: Supplementary file 17 — Figure S17. Lymph node metastasis of mice subcutaneously injected with LuTDNa3 cell cultures. A. Sections of H&E stained lymph nodes of mice injected with LuTDco or LuTDNa3 cell cultures showed the presence of, respectively, infrequent atypical cells lacking differentiation features (top panels), and prominent differentiated metastatic lesions (bottom panels). B. Lymph node sections were stained for eGFP and this confirmed that the metastatic lesions in the lymph nodes of the LuTDNa3-injected mice were derived from the transgene-positive primary tumors, as expected. Panels correspond to increasing magnification from left to right. Bars, 50 μm. (PDF 7298 kb) [file 12885_2019_5807_MOESM17_ESM.pdf]

A.

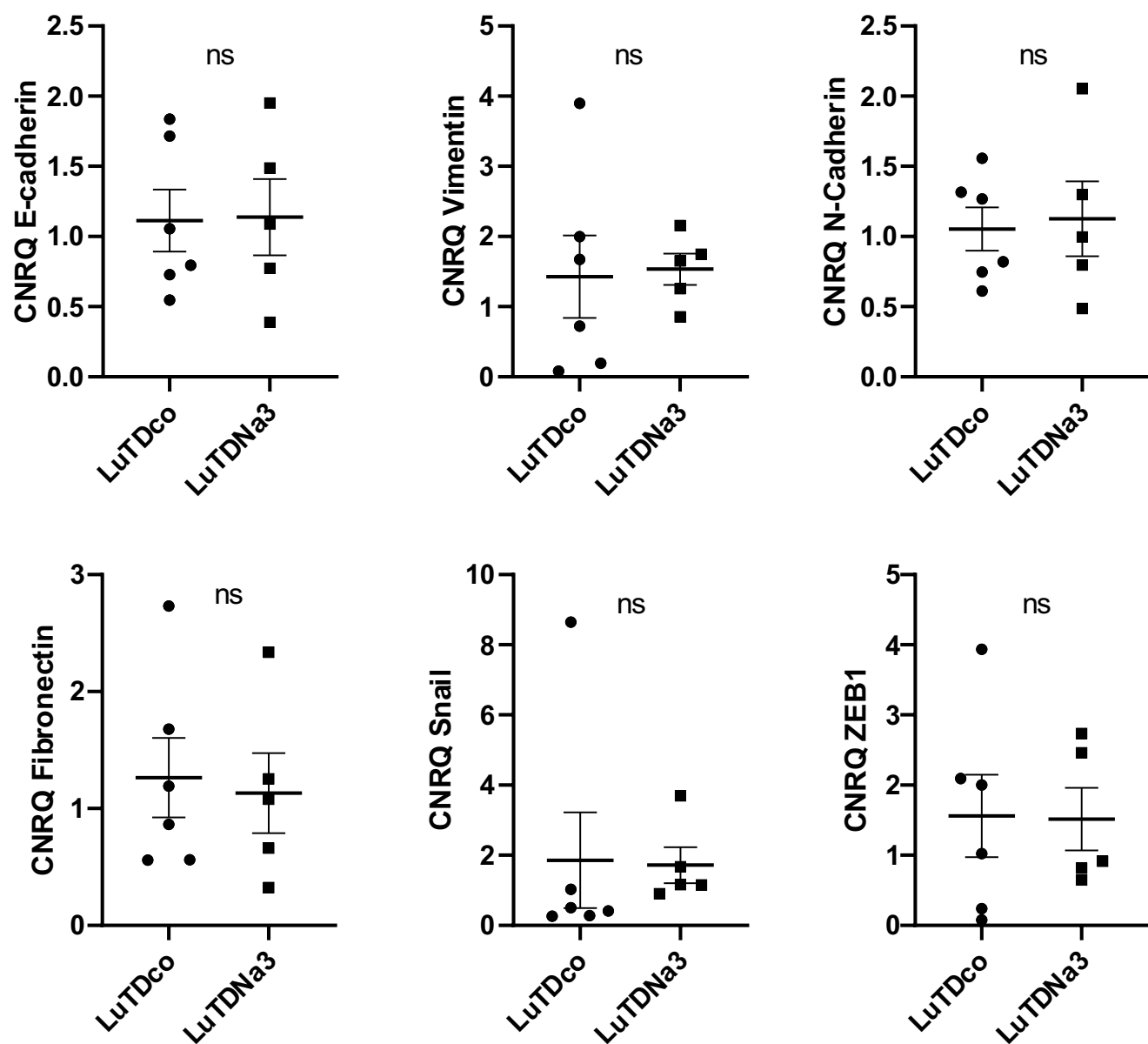

B.

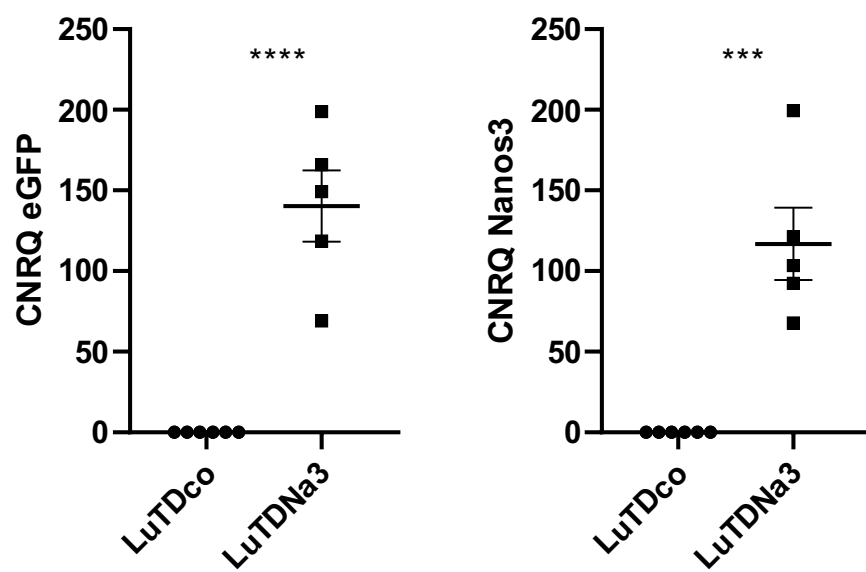

Supplement: Supplementary file 18 — Figure S18. EMT is not involved in the increased lymph node metastasis by LuTDNa3 cells. A. Expression analyses of EMT-related genes by qRT-PCR of transcripts in primary lung tumor-derived cell lines LuTDco and LuTDNa3. This experiment revealed no significant differences between LuTDco and LuTDNa3 cell lines for the expression levels of the following genes, Cdh1, Vim, Cdh2, Fn, Snai1 and Zeb1. B. Expression analyses of eGFP and Nanos3 by qRT-PCR in primary lung tumor-derived cell lines LuTDco and LuTDNa3 showed specific eGFP and Nanos3 expression in LuTDNa3 cell lines. CNRQ, calibrated normalized relative quantity, error bars, SEM, ***: P ≤ 0.001 and ****: P ≤ 0.0001 Gene expression was normalized to reference genes (eef1a and hmbs) using qbase+ (Biogazelle) [35]. (PDF 1358 kb) [file 12885_2019_5807_MOESM18_ESM.pdf]

**A.**

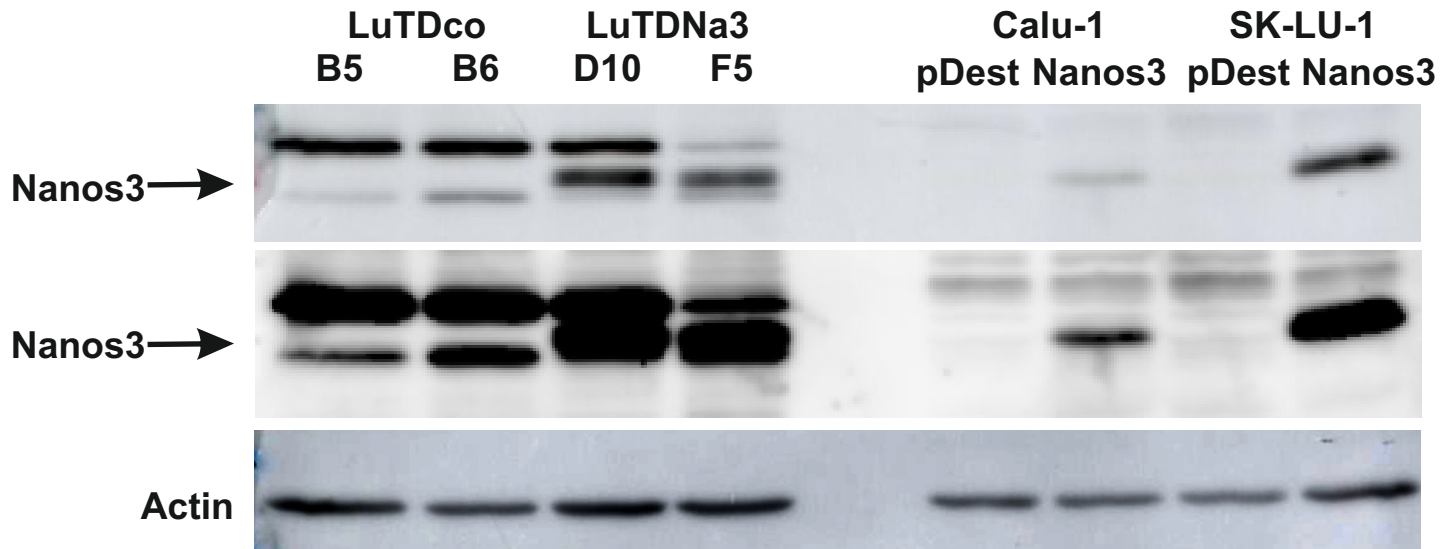

**B.**

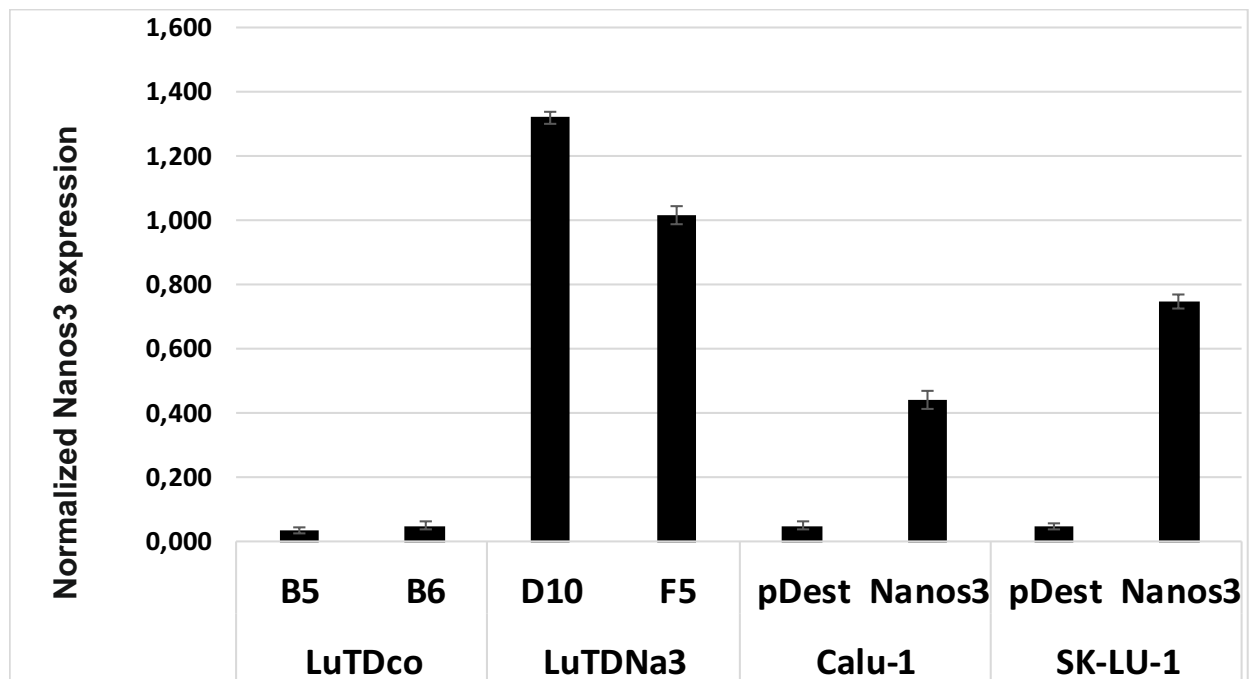

Supplement: Supplementary file 19 — Figure S19. Comparison of Nanos3 expression levels in lung tumor-derived mouse cell lines and established human lung cancer cell lines. A. Protein levels of Nanos3 were detected by western blotting. Top and middle panel represent lower and higher exposure time, respectively, for Nanos3 detection. The Nanos3-specific bands are indicated by the arrows. Actin expression acted as a loading control (bottom panel). B. Quantification of Nanos3 levels in the blot of (A), normalized against actin signals. (PDF 1411 kb) [file 12885_2019_5807_MOESM19_ESM.pdf]
