# Supplementary material for: A new mouse model to study the role of ectopic Nanos3 expression in cancer
Source: BMC Cancer. 2019 Jun 17;19:598. doi: 10.1186/s12885-019-5807-x (PMC6580527; doi:10.1186/s12885-019-5807-x)

Focal hyperplasia

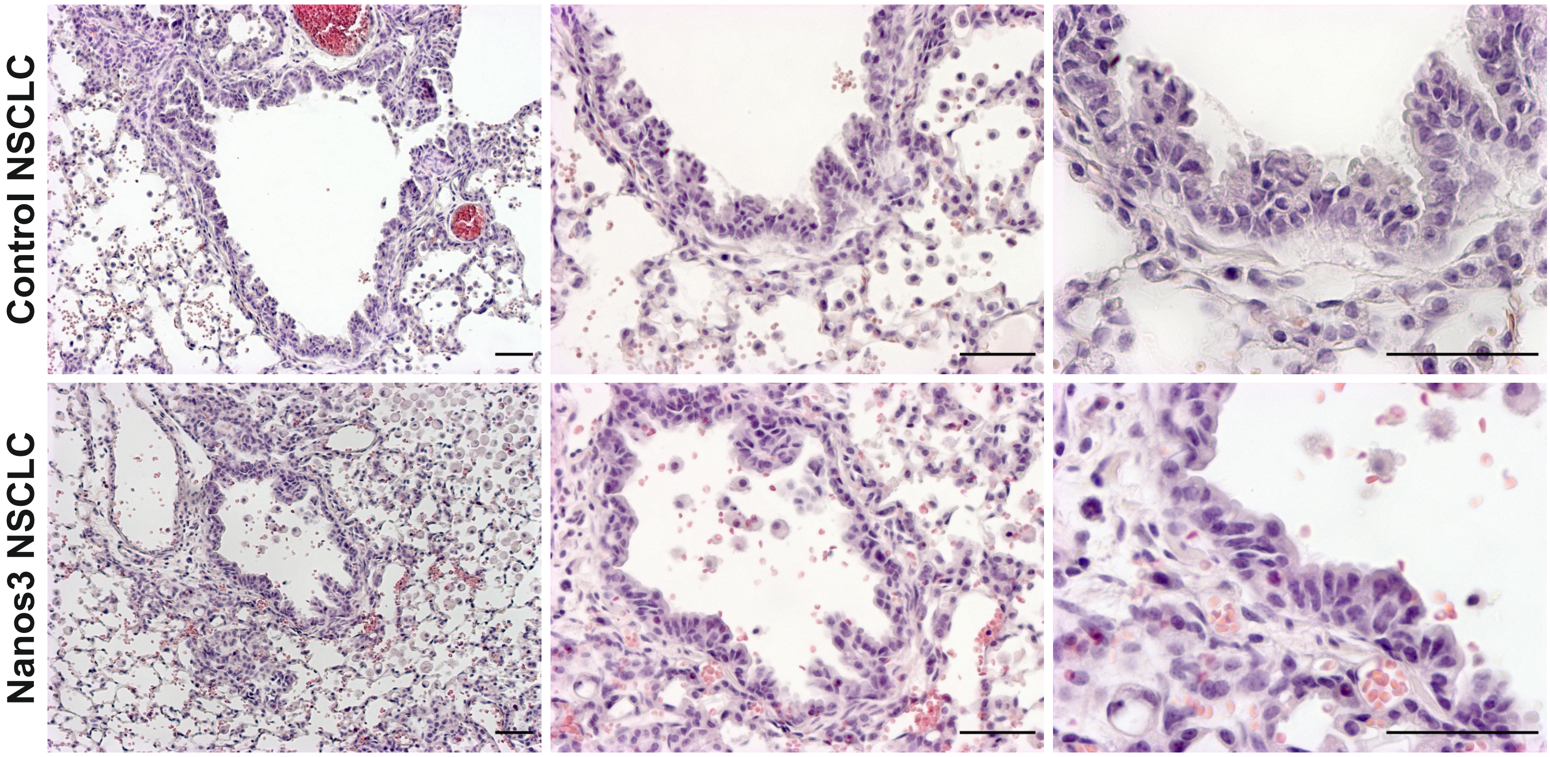

Papillary hyperplasia

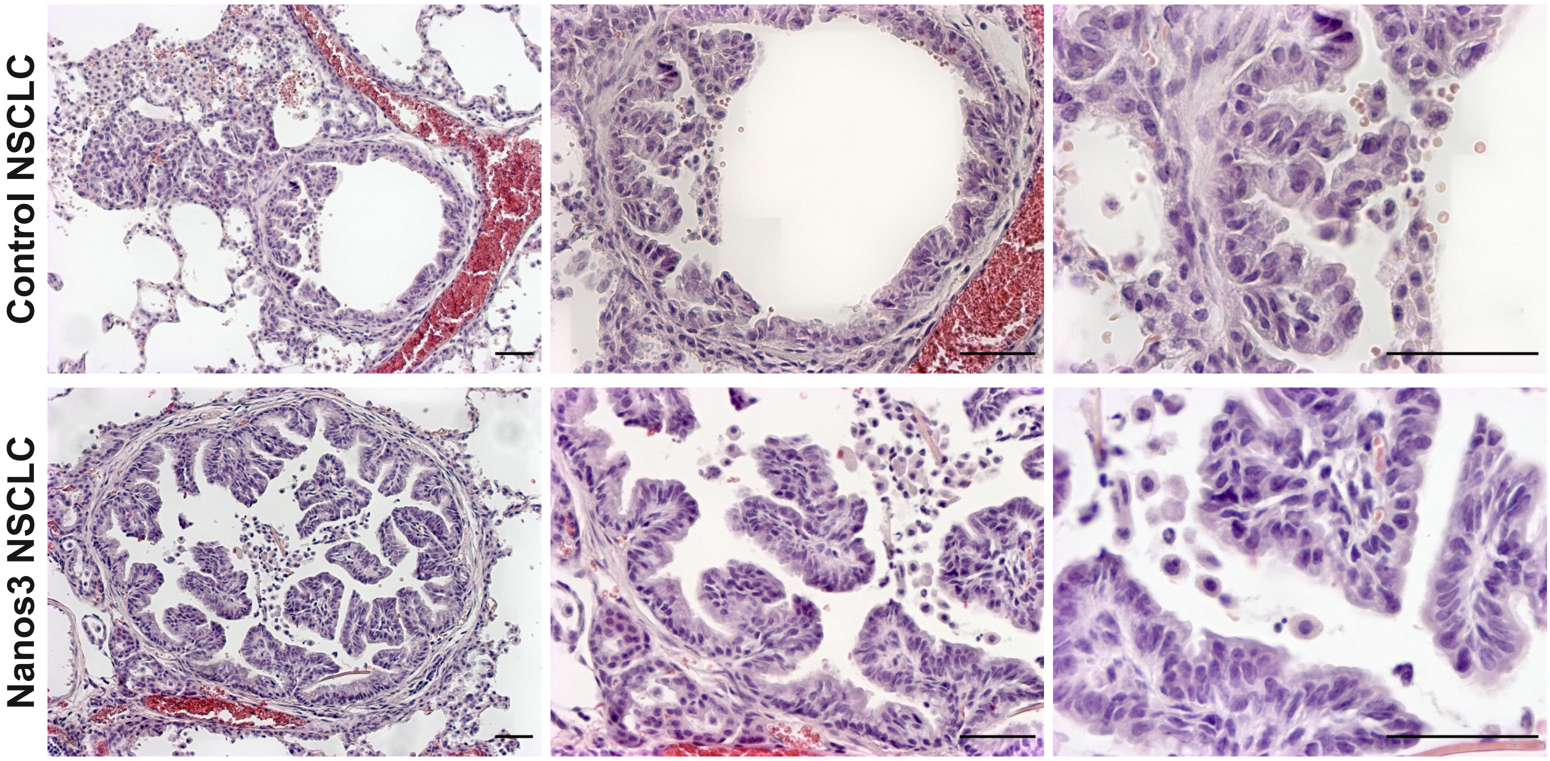

Supplement: Supplementary file 7 — Figure S7. Microscopic images of H&E-stained lung sections from control and Nanos3 NSCLC mice show different stages of tumor progression in the bronchiolar tissue. Focal and papillary hyperplasia were observed in the bronchioles of both control and Nanos3 NSCLC mice. Panels correspond to increasing magnification from left to right. Bars, 50 μm. (PDF 6768 kb) [file 12885_2019_5807_MOESM7_ESM.pdf]
