# Supplementary material for: A new mouse model to study the role of ectopic Nanos3 expression in cancer
Source: BMC Cancer. 2019 Jun 17;19:598. doi: 10.1186/s12885-019-5807-x (PMC6580527; doi:10.1186/s12885-019-5807-x)
Supplement: Supplementary file 20 — Table S1. A list of the primers used for genotyping. (DOCX 14 kb) [file 12885_2019_5807_MOESM20_ESM.docx]

**Additional file 20: Table S1. A list of the primers used for genotyping.**

| **Allele** | **Primer name** | **Primer sequence** |
| --- | --- | --- |
| **Rosa floxed (Nanos3 mouse)** | G1 | 5’-TAGGTAGGGGATCGGGACTCT-3’ |
|  | G2 | 5’-GCGAAGAGTTTGTCCTCAACC-3’ |
| **Cre** | Cre_F | 5'-TGCCACGACCAAGTGACAGCAATG-3' |
|  | Cre_R | 5'-AGAGACGGAAATCCATCGCTCG-3' |
| **Nanos3-IRES-eGFP** | Nanos3_F | 5'-AAGGATCAGAAGCGCAGCCT-3' |
|  | Nanos3_R | 5'-GGGCGGAATTCGATATCAAG-3' |
| **Rosa** | Rosa_F | 5'-AAAGTCGCTCTGAGTTGTTAT-3' |
|  | Rosa_R1 | 5'-GGAGCGGGAGAAATGGATATG-3' |
|  | Rosa_R2 | 5'-GCGAAGAGTTTGTCCTCAACC-3' |
| **Nanos3 deletion** | Nanosdel_F | 5'-GGCGCAGTAGTCCAGGGTTTCCTTG-3' |
|  | Nanosdel_R1 | 5'-AGCCGGTTGGCGCTACCGGT-3' |
|  | Nanosdel_R2 | 5'-GCCGTTGTGTTTGCAGAAAGAGCA-3' |
| **LSL-KRas^G12D^** | KRas_F | 5'-ATGTCTTTCCCCAGCACAGT-3' |
|  | KRas_R1 | 5'-TCCGAATTCAGTGACTACAGATG-3' |
|  | KRas_R2 | 5'-CTAGCCACCATGGCTTGAGT-3' |
| **CCSP-rtTA** | CCSP-rtTA_F | 5'-AAAATCTTGCCAGCTTTCCCC-3' |
|  | CCSP-rtTA_R | 5'-ACTGCCCATTGCCCAAACAC-3' |
| **p53** | p53_F | 5'-AAGGGGTATGAGGGACAAGG-3' |
|  | p53_R | 5'-GAAGACAGAAAAGGGGAGGG-3' |
