# Supplementary material for: A new mouse model to study the role of ectopic Nanos3 expression in cancer
Source: BMC Cancer. 2019 Jun 17;19:598. doi: 10.1186/s12885-019-5807-x (PMC6580527; doi:10.1186/s12885-019-5807-x)
Supplement: Supplementary file 23 — Table S2. A list of the RT-qPCR primers used. (DOCX 14 kb) [file 12885_2019_5807_MOESM23_ESM.docx]

**Additional file 23: Table S2. A list of the RT-qPCR primers used.**

| **Gene name** | **Forward primer (5'-3')** | **Reverse primer (5'-3')** |
| --- | --- | --- |
| ***eGFP*** | GAGCTGAAGGGCATCGACTT | TCTGCTTGTCGGCCATGAT |
| ***NANOS3* (1)** | CGCAAACACAACGGCGAGTC | CGGGTGGTGTGGCTGTAGA |
| ***NANOS3* (2)** | CAGGGCTACACCTCCGTCTACA | ACTTCCCGGCACCTCTGAAACC |
| ***Cdh1*** | TGCCATCCTCGGAATCCTT | GGCTCTTTGACCACCGTTCTC |
| ***Cdh2*** | CCAGGTTTGGAATGGGTCTGT | GTACCGCAGCATTCCATTCA |
| ***Fn1*** | AGACTCGAGGCGGAAATTCC | CCCTGCGACCCTCAGAAGT |
| ***Snai1*** | CGGAAGCCCAACTATAGCGA | GGTCGTAGGGCTGCTGGAA |
| ***Vim*** | TGGTTGACACCCACTCAAAA | GGTCATCGTGATGCTGAGAA |
| ***Zeb1*** | TTGCGTGTCAGGCATGGAT | GAAAACGGCTGTGAACCAAAA |
| ***Tbp*** | TCTACCGTGAATCTTGGCTGTAAA | TTCTCATGATGACTGCAGCAAA |
| ***Hmbs*** | GAAACTCTGCTTCGCTGCATT | TGCCCATCTTTCATCACTGTATG |
| ***ActB*** | GCTTCTAGGCGGACTGTTACTGA | GCCATGCCAATGTTGTCTCTTAT |
| ***Hprt1*** | AGTGTTGGATACAGGCCAGAC | CGTGATTCAAATCCCTGAAGT |
| ***Rpl13A*** | CCTGCTGCTCTCAAGGTTGTT | TGGTTGTCACTGCCTGGTACTT |
| ***Sdha*** | CTTGAATGAGGCTGACTGTG | ATCACATAAGCTGGTCCTGT |
| ***Ywhaz*** | TAAATGGTCTGTCACCGTCT | GGAAATACTCGGTAGGGTGT |
